# Structural analysis of the SRP Alu domain from *Plasmodium falciparum* reveals a non-canonical open conformation

**DOI:** 10.1038/s42003-021-02132-y

**Published:** 2021-05-20

**Authors:** Komal Soni, Georg Kempf, Karen Manalastas-Cantos, Astrid Hendricks, Dirk Flemming, Julien Guizetti, Bernd Simon, Friedrich Frischknecht, Dmitri I. Svergun, Klemens Wild, Irmgard Sinning

**Affiliations:** 1 Heidelberg University Biochemistry Center (BZH), Heidelberg, Germany; 2 European Molecular Biology Laboratory (EMBL), Hamburg, Germany; 3 Integrative Parasitology, Center for Infectious Diseases, Heidelberg University Hospital, Heidelberg, Germany; 4 European Molecular Biology Laboratory (EMBL), Heidelberg, Germany

**Keywords:** Solution-state NMR, SAXS

## Abstract

The eukaryotic signal recognition particle (SRP) contains an Alu domain, which docks into the factor binding site of translating ribosomes and confers translation retardation. The canonical Alu domain consists of the SRP9/14 protein heterodimer and a tRNA-like folded Alu RNA that adopts a strictly ‘closed’ conformation involving a loop-loop pseudoknot. Here, we study the structure of the Alu domain from *Plasmodium falciparum* (*Pf*Alu), a divergent apicomplexan protozoan that causes human malaria. Using NMR, SAXS and cryo-EM analyses, we show that, in contrast to its prokaryotic and eukaryotic counterparts, the *Pf*Alu domain adopts an ‘open’ Y-shaped conformation. We show that cytoplasmic *P. falciparum* ribosomes are non-discriminative and recognize both the open *Pf*Alu and closed human Alu domains with nanomolar affinity. In contrast, human ribosomes do not provide high affinity binding sites for either of the Alu domains. Our analyses extend the structural database of Alu domains to the protozoan species and reveal species-specific differences in the recognition of SRP Alu domains by ribosomes.

## Abstract (web summary)

The SRP Alu domain is involved in co-translational protein targeting. Soni et al. investigate the Alu domain of *Plasmodium falciparum*, the agent of malaria, and find that unlike any other it adopts an open conformation.

## Introduction

Malaria is caused by the apicomplexan protozoan *Plasmodium falciparum*, which exhibits a complex two-host life cycle including a sexual stage in the mosquito and an asexual blood stage in humans. Given the importance of protein synthesis at the asexual blood stages of the parasite, its protein translation machinery has been an important target for the development of many anti-malarial drugs^1^. *P. falciparum* has a complex three-compartment protein translation machinery with the majority of translation carried out by cytoplasmic ribosomes^2^ and a smaller subset by the prokaryotic-like organellar ribosomes of mitochondria and the non-photosynthetic apicoplast^1^. The complexity of its protein translation machinery extends to the immense task of protein trafficking of secretory proteins important for invasion and adhesion of infected red blood cells (RBCs)^3,4^. Several secretory pathways tackle protein trafficking, and among them co-translational protein targeting to the endoplasmic reticulum (ER) is mediated by the classical signal recognition particle (SRP)^5^.

**Fig. 1:**
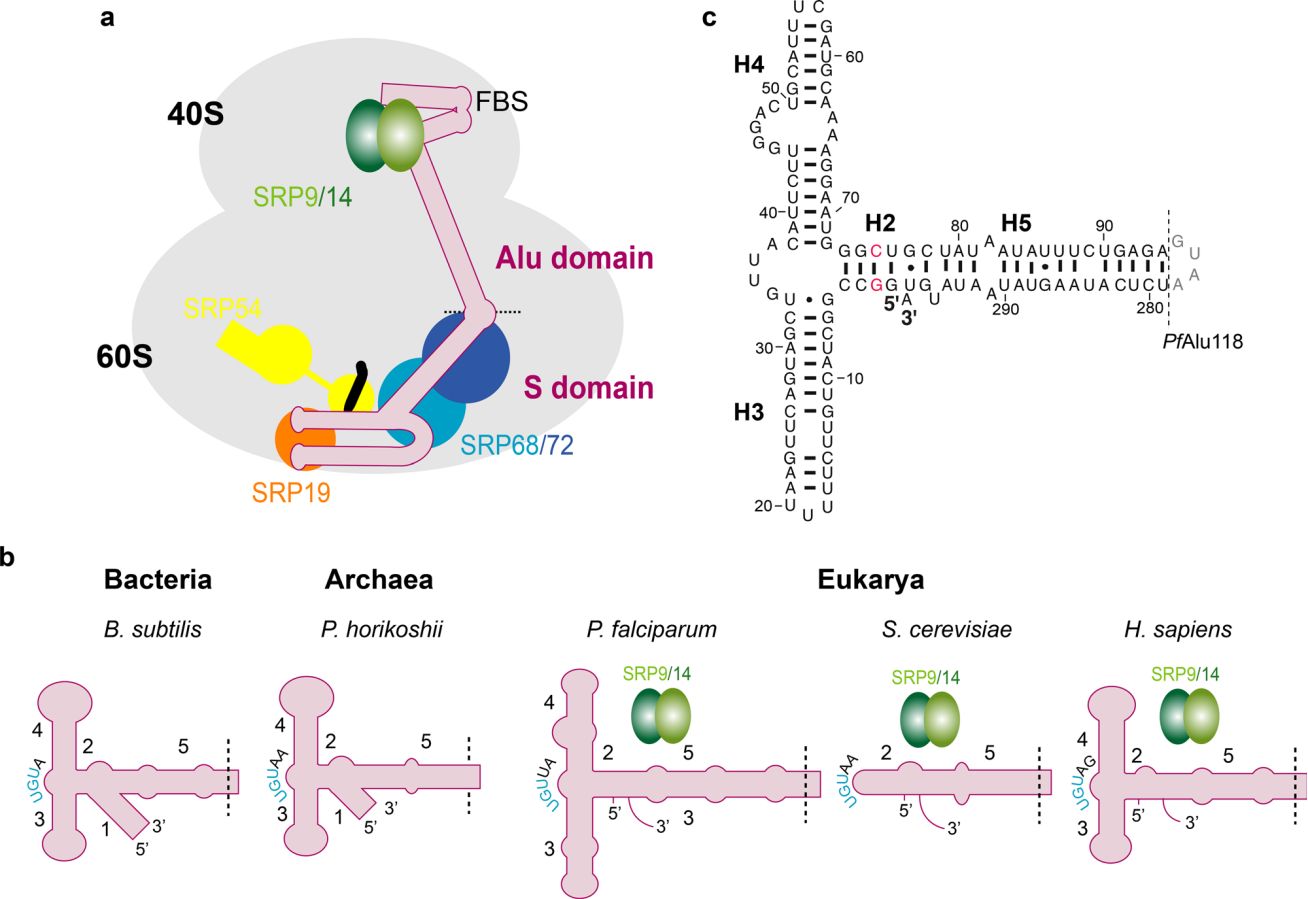
Architecture of the SRP Alu domain. **a** Schematic showing the composition of the eukaryotic SRP bound to the ribosome-nascent chain complex (RNC). **b** Schematic representation of SRP Alu RNA from the three domains of life: Bacteria, archaea, and eukarya. SRP9/14 proteins are shown for organisms where they have been annotated. A dashed line indicates the border between the Alu and S domains. The conserved UGU motif is colored in blue. **c** Construct for the entire *Pf*Alu RNA (*Pf*Alu118) where the S domain is replaced by a GUAA tetraloop (gray). Base-pair information is depicted as previously predicted^8^. Nucleotides mutated due to the hammerhead ribozyme and T7 RNA polymerase production requirements are highlighted in red.

SRP is an elaborate molecular machine that co-translationally recognizes hydrophobic N-terminal signal sequences or transmembrane domains as they emerge from the translating ribosome (ribosome-nascent chain complex, RNC). The eukaryotic SRP is a ribonucleoprotein complex assembled onto an elongated ~300 nucleotide (nt) 7SL RNA (in the following denoted as SRP RNA). It is divided into two distinct functional fragments known as the 5′ Alu and 3′ S domains, which are connected by a hinge and span from the inter-subunit interface to the tunnel exit of the ribosome^6^. The S domain contains proteins SRP19, SRP54, and SRP68/72 and is involved in signal recognition and receptor targeting, whereas the Alu domain comprises the SRP9/14 heterodimer and interferes with translation elongation^6,7^ (Fig. 1a). All these components of the eukaryotic SRP have also been identified in the malarial parasite^8–10^. Colocalization studies performed in asexual blood stages of the malarial parasite show that SPR9/14 is associated with SRP19 and SRP54, demonstrating the in vivo existence of SRP^10^. In addition to being a central component of the SRP machinery, the SRP RNA is also the precursor of retrotransposable Alu elements that represent ~11% of the human genome^11^. It was recently shown that a minimal functional Alu-retrotransposon (Alu-RT) retains SRP9/14-binding capacity^12^.

The SRP Alu domain is known to increase translocation efficiency by preventing premature release of the nascent chain before proper engagement of the RNC with the translocation machinery at the ER^13^. For *Bacillus subtilis*, a “dock-and-block” mechanism was established, whereby the Alu domain docks into the factor binding site (FBS) of the RNC to block translation elongation^14^. Although elongation arrest is not a prerequisite for protein targeting in vitro^15^, its abrogation causes significant growth defects and protein translocation defects in mammalian^16^, mammalian-heterologous^17^, and yeast-homologous translation/translocation systems^18^. However, ribosome-profiling studies in *Saccharomyces cerevisiae* suggest substantial diversity concerning elongation arrest activity of the SRP Alu domain^19^.

In cryo-electron microscopy (cryo-EM) reconstructions of mammalian SRP bound to RNCs, the Alu domain shows a bipartite mode of interaction^6,20^; whereas the Alu RNA forms contacts with protein uL11 of the large ribosomal 60S subunit at the L7/L12 stalk, the SRP9/14 heterodimer interacts with the 5′ domain (helices h5, h14, and h15) of 18S rRNA within the small ribosomal 40S subunit^6,20^ (Supplementary Fig. 1). Further, recent data suggest that only the S domain binds with high affinity to RNCs^21^ where it is responsible for scanning the signal sequences, whereas the Alu domain can swing away from the RNCs only engaging with the FBS when retardation of translation is required for targeting^20^.

The universally conserved SRP has acquired a variety of substantial adaptations during the course of evolution. For instance, Gram-negative bacteria like *Escherichia coli* only possess a minimal SRP, consisting of a 4.5 S RNA and SRP54 and completely lacking the Alu domain. In contrast, gram-positive bacteria such as *B. subtilis* contain the Alu RNA but lack the SRP9/14 heterodimer. Archaea and most eukaryotes contain a large SRP RNA with additional secondary structural elements in the Alu RNA (helices H1 to H5)^22,23^ (Fig. 1b). The Alu RNA itself is divided into a 5′ domain (up to helix H4) and a 3′ domain consisting of helix H5. Considerable variations also exist in the SRP Alu RNA of lower eukaryotes; fungi such as *S. cerevisiae* (Fig. 1b), *Encephalitozoon cuniculi*, and *Saccharomyces kluyveri* completely lack helices H3 and H4 while certain euglenozoa including *Trypanosoma brucei* contain shortened helices^8^. In *T. brucei*, the presence of a tRNA-like molecule has been suggested to compensate for the lack of a “proper” Alu domain^24,25^. Even more diversity exists within alveolates, where organisms such as *P. falciparum* and *Plasmodium knowlesi* contain considerable extensions in helices H3 and H4, whereas, e.g., *Tetrahymena rostrata* and *Theileria annulata* contain a short helix H3 or completely lack helix H4, respectively.

Despite these variations in the composition of the Alu domain, the comparison of crystal structures of the Alu domain from bacteria (*B. subtilis*)^26^, archaea (*Pyrococcus horikoshii*)^27^, eukarya (*Homo sapiens*)^28^, and the Alu-RT complex^12^ shows a substantial degree of conservation in the overall architecture of the Alu domain. In our current structural understanding of a canonical eukaryotic SRP Alu domain the 3′ domain of the RNA folds back on the 5′ domain like a jack-knife^28^, exposing a central conserved UGU motif that forms a U-turn (Supplementary Fig. 2). This U-turn positions helices H3 and H4 such that their apical loops base pair to form a loop–loop pseudoknot (Supplementary Fig. 2), which is a conserved feature present in all SRP Alu domain crystal structures^12,26–28^. The loop–loop pseudoknot is necessary for the SRP Alu RNA to fold into a tRNA-like conformation and mutations in either loop of the human Alu RNA result in Alu domain assembly defects^29^. The UGU motif is recognized by the SRP9/14 proteins, whose binding determines the orientation of the 5′ and 3′ domains at the three-helix junction and stabilizes the overall fold of the Alu domain^28,29^. However, in bacteria and archaea, the lack of SRP9/14 is compensated for by the addition of helix H1, which provides rigidity at the three-helix junction (Fig. 1b). In addition, the loop–loop pseudoknot is extended, comprising five continuous base pairs as opposed to three in the case of the human Alu domain^26–28^.

Comparison of the sequences and secondary structure predictions of *P. falciparum* (*Pf*Alu) and human (*Hs*Alu) RNA indicates that helices H3 and H4 of *Pf*Alu RNA contain insertions and have smaller apical loops^8^ (Supplementary Fig. 3a). Here, we use a combination of nuclear magnetic resonance (NMR) spectroscopy, small-angle X-ray scattering (SAXS), and cryo-EM to study the direct impact of these modifications in helices H3/H4 on the assembly, flexibility, and structure of the *Pf*Alu domain. We show that the *Pf*Alu RNA is structurally divergent from its eukaryotic counterparts, thereby challenging the “dock-and-block” mechanism of the Alu domain at the ribosomal FBS. Furthermore, we study the ribosome-binding properties of the *Pf*Alu domain using microscale thermophoresis (MST) and provide insights into its distinct interactions with the ribosome.

## Results

### Purification and assembly of *Pf*Alu domain

**Fig. 2:**
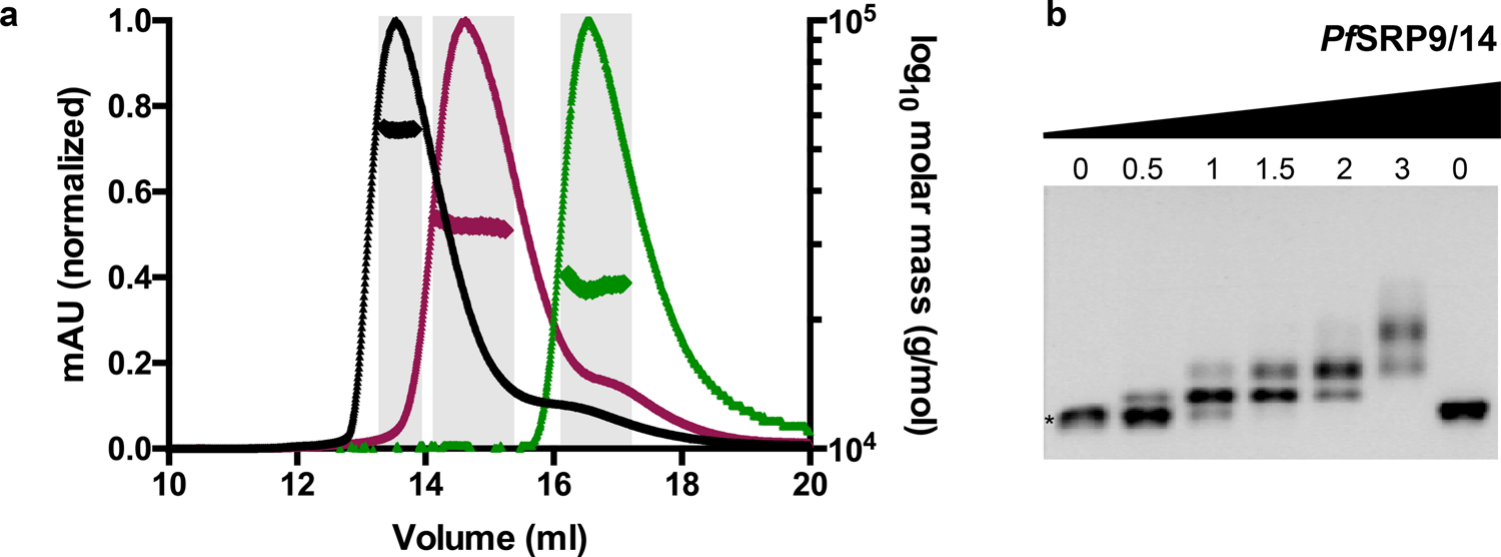
Assembly of the *Pf*Alu domain. **a** Profiles of size-exclusion chromatography coupled to multi-angle light scattering of *Pf*Alu118 RNA (magenta), *Pf*SRP9/14 (green), and the complete *Pf*Alu domain comprising *Pf*Alu118 RNA and *Pf*SRP9/14 (black) are shown. Molar mass distribution across the respective peaks is marked and fractions used for obtaining an average molar mass are highlighted in gray. **b** EMSA showing quantitative shifts of the *Pf*Alu118 RNA upon titration with increasing amounts of *Pf*SRP9/14. The asterisk (*) indicates unbound RNA. The molar-fold excess of *Pf*SRP9/14 over *Pf*Alu118 RNA is indicated on the top. The uncropped gel image is included in Supplementary Data 1.

In order to understand the molecular assembly of the *Pf*Alu domain, we first purified the RNA and protein components separately, then monitored their assembly in vitro. A 118 nt *Pf*Alu RNA (henceforth named *Pf*Alu118) harboring the complete Alu RNA was generated by replacing the S domain with a GUAA tetraloop in helix H5 (Fig. 1c). The RNA was produced by in vitro transcription and subsequently refolded by snap cooling. After refolding, size-exclusion chromatography (SEC) coupled to multi-angle light scattering experiments (SEC-MALS) showed that the RNA was a homogenous monomer (Fig. 2a). Co-expression and co-purification of *Pf*SRP9/14 proteins also yielded a highly pure heterodimer, as shown by uniform molecular weight distribution in SEC-MALS experiments (Fig. 2a, Supplementary Table 1). Next, the complete *Pf*Alu domain was assembled. A marked change in the retention volume of the *Pf*Alu domain compared to the free *Pf*Alu118 RNA and *Pf*SRP9/14 heterodimer during gel filtration, together with SEC-MALS experiments, validated the formation of the Alu domain complex (Fig. 2a, Supplementary Table 1). Consistently, gel shifts were observed upon titration of the *Pf*SRP9/14 heterodimer with the free *Pf*Alu118 RNA in electrophoretic mobility shift assays (EMSAs) (Fig. 2b). These data show that the complete *Pf*Alu domain can be assembled to the homogeneity required for detailed structural and functional studies. In order to understand the architecture of the *Pf*Alu domain, we first started with a crystallographic approach. After several rounds of optimization with a variety of crystallization carriers, we were able to obtain crystals, which however showed poor X-ray diffraction that could not be improved. Therefore, we combined NMR spectroscopy, SAXS, and cryo-EM to understand the structure of the *Pf*Alu domain.

### NMR analyses of *Pf*Alu118 RNA

Imino resonances are only present in guanine and uracil bases. They are highly sensitive to solvent exchange and indicative of solvent protection owing to their involvement in base-pairing (reviewed in ref. ^30^). Therefore, the assignment of imino resonances is helpful in RNA secondary structure determination. For *Pf*Alu118 RNA^8^, secondary structure predictions suggest that it harbors longer helices H3 and H4 compared with *Hs*Alu RNA (Supplementary Fig. 3a). We, therefore, used NMR to determine base-pairing within *Pf*Alu118 RNA and validate its secondary structure prediction. Imino–imino cross-signals observable in two-dimensional nuclear overhauser effect spectroscopy (NOESY) experiments are useful for the identification and sequential assignment of imino proton resonances. However, the severe spectral overlap due to low chemical diversity of the building blocks of RNA coupled with its shorter transverse relaxation times can make such NMR analyses quite challenging for large RNAs^31–33^. We, therefore, first recorded imino one-dimensional spectra of a shorter 76 nt RNA (henceforth, referred to as *Pf*Alu76 RNA) lacking helix H5 (Supplementary Fig. 3a). *Pf*Alu76 RNA was still relatively large in size for NMR measurements, with significant overlap and broadening of imino resonances. Therefore, we used a divide-and-conquer approach towards resonance assignment, breaking down the *Pf*Alu118 RNA into individual helices H3 (30 nt), H4 (41 nt), and H5 (43 nt) (Supplementary Fig. 3b).

**Fig. 3:**
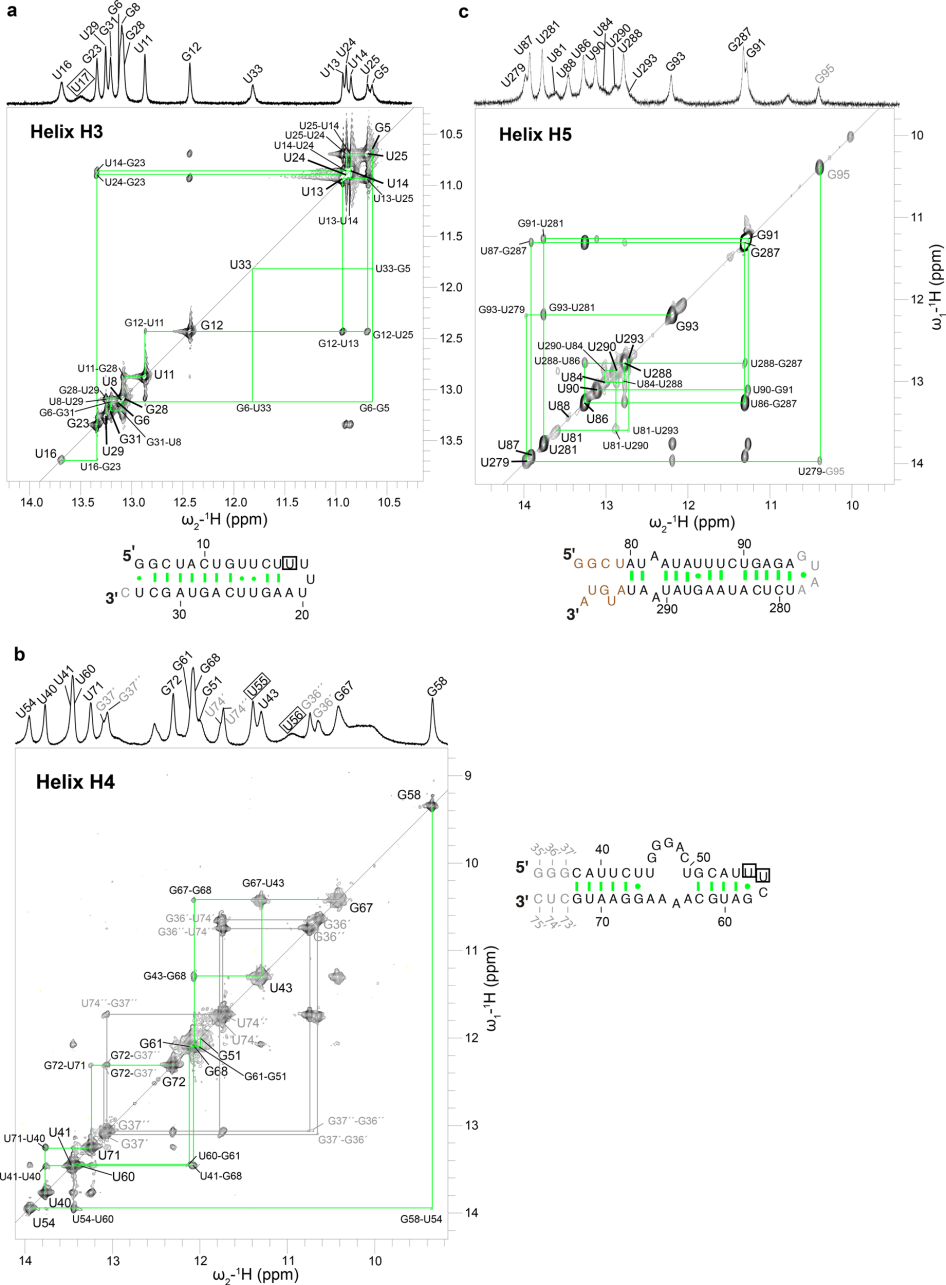
*Pf*Alu RNA secondary structure. Imino regions of 1D and 2D ^1^H,^1^H-NOESY spectra of helices H3, H4, and H5 are shown in **a**–**c**, respectively. RNA sequences of the corresponding helices are also shown. Nucleotides colored in gray have been added artificially to the Alu RNA while those in brown could not be assigned. Wobble base pairs are connected by a dot while canonical Watson–Crick base pairs are connected by a hyphen. Resonances, which did not give imino cross-peaks to neighboring bases and were assigned by exclusion, are boxed. Helix H4 resonances exhibiting alternative conformations are numbered in gray.

We recorded two-dimensional ^1^H,^1^H imino-NOESY spectra of helices H3, H4, and H5 separately (Fig. 3). The canonical Watson–Crick base pairs range from 12 to 13.5 ppm for G-C and 13–15 ppm for U-A, whereas G•U and U•U wobble base pairs occur at ~10–12 ppm and ~10.4–11.3 ppm, respectively. The wobbles are easily identified due to the strong nuclear overhauser effect (NOE) cross-peaks between base-paired uracil and/or guanine imino protons^30^. Here, sequential imino–imino NOE cross-peaks were used to assign the fingerprint spectra for all three helices. Strong cross-peaks between two wobble pairs (U13•U25 and U14•U24) served as the starting point for resonance assignment in helix H3 (Fig. 3a). The formation of these wobble base pairs suggests the absence of the predicted internal symmetric loop^8^ in helix H3, which displays continuous stacking in this region instead. Imino protons of U14•U24 and U13•U25 gave strong cross-peaks to G23 and G12, respectively, and that of G23 gave a weak cross-peak to the imino proton of U16. On the other side of the U13•U25 wobble base pair, sequential connectivity between G12, U11, G28, U29, U8, G31, and G6 could be established unambiguously. The closing base pair of the stem G5•U33 gave extremely weak diagonal peaks as well as cross-peaks to G6. The identity of this G•U base pair was established by the average chemical shift values reported for a canonical G•U wobble (U33 at ~11.8 ppm, G5 at ~10.6 ppm). The only imino proton that did not give a signal in the 2D-NOESY was assigned to U17 and imino protons of U18 and U20 could not be assigned due to lack of peaks in the 1D spectrum, indicating that they are solvent accessible.

In a similar approach, upfield chemical shift characteristic of guanine in a tetraloop^30^ aided in the identification of G58 (~9.35 ppm) within the ^55^UUCG^58^ tetraloop. It served as the starting point for resonance assignment of helix H4 (Fig. 3b) and sequential connectivity between G58, U54, U60, G61, and G51 was established. The imino protons from closing base pairs of the predicted internal asymmetric loop in H4 are not visible (U50, U44), indicating that they are solvent accessible. Strong NOE cross-peaks between imino protons of the U43•G67 wobble is observed, which helped in the assignment of neighboring G68, U41, U40, and U71 unambiguously. Next, two sets of sequential connections lead to U71 and are indicative of alternative conformations existing in this region of H4: G72–G37’–G36’–U74’; G72–G37”–G36”–U74”. This alternative conformation is likely non-physiological as these base pairs are not present in the wild-type SRP Alu RNA, and have been artificially introduced owing to template sequence requirements for in vitro transcription or for 3′-hammerhead cleavage. The diagonal peaks for imino protons of U55, U56, and G35′ are not visible in the 2D-NOESY owing to fast water exchange, therefore resonance assignments of U55, U56 were inferred by exclusion and from previously published chemical shifts of imino protons from bases in a tetraloop^30^. We do not observe any imino protons belonging to residues U44–U50, consistent with the prediction of an internal asymmetric loop.

Next, resonance assignment of helix H5 was started from the central U86•G287 wobble pair, which shows strong imino cross-peaks to each other (Fig. 3c). On one side of the U86•G287 wobble, sequential imino correlations could be observed for bases U288, U84, U290, U81, and U293. On the other side, however, correlations could only be observed to the U87 imino proton. Another stretch of bases with strong imino–imino cross-peaks aided in the unambiguous assignment of U90, G91, U281, G93, and U279. Upfield chemical shift characteristic of guanine in a tetraloop^30^, coupled with a weak cross-peak to the imino proton of U279 helped in assignment of G95. Although no imino cross-peak between the sequential neighbors U87 and U88 was visible, cross-peaks between the A286 H2 proton and U88 imino group and the A285 H2 proton and U87 aided in assignment of the U88 imino proton (Supplementary Fig. 4). Imino protons of two more bases (at 10.01 ppm and 12.07 ppm) are visible but the lack of imino cross-peaks to sequential neighbors impeded their unambiguous assignment. Similarly, the peak at 10.8 ppm in the 1D NMR spectrum could not be assigned.

In total, the imino resonances revealed the presence of 12, 11, and 14 stable hydrogen bonds in helices H3, H4, and H5 of *Pf*Alu118 RNA, respectively, and provided experimental validation of the predicted secondary structure of all helices.

### SAXS analyses of *Pf*SRP9/14 heterodimer and the *Pf*Alu118 RNA

**Fig. 4:**
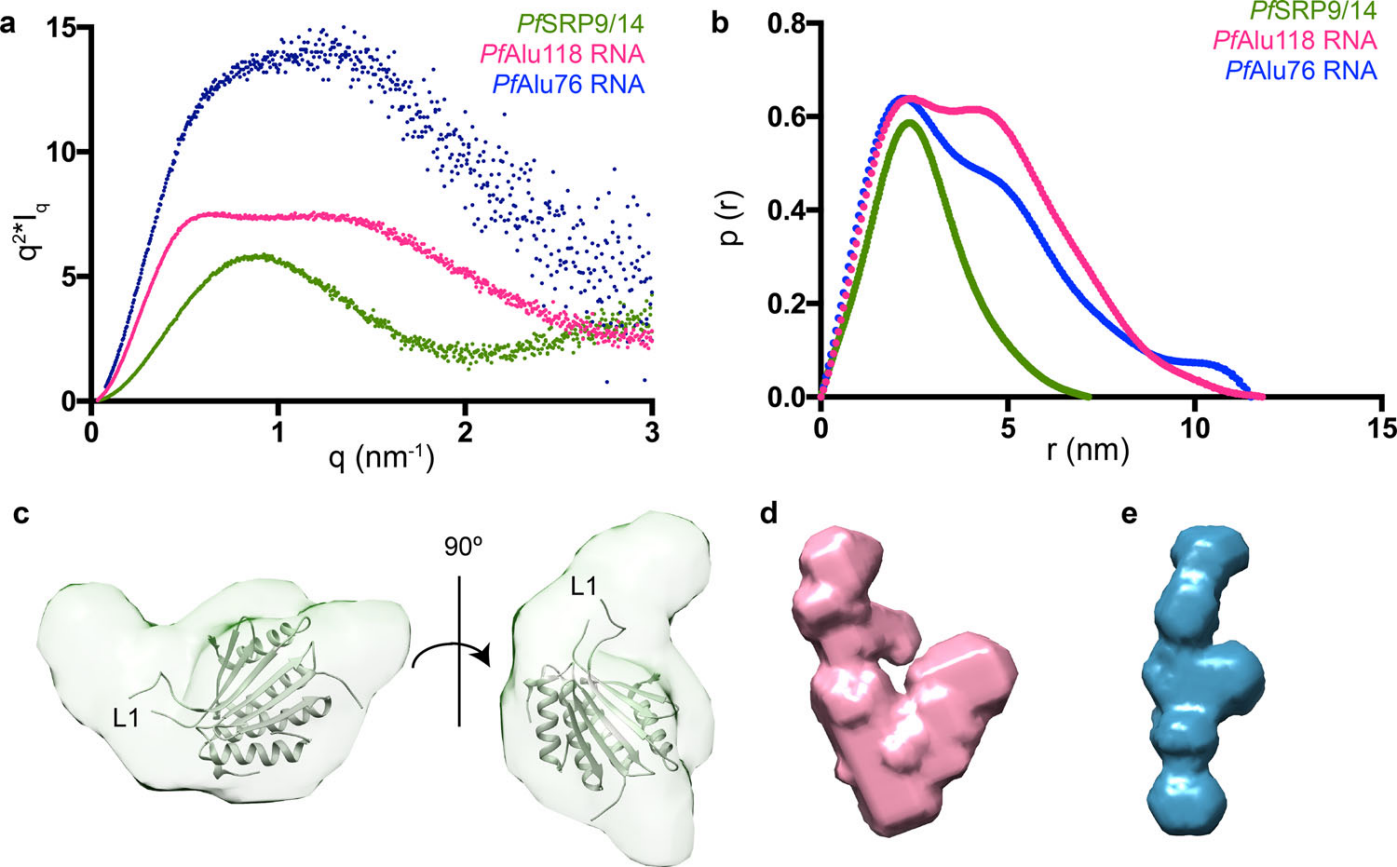
SAXS analyses of the *P. falciparum* Alu domain protein and RNA components. **a** Kratky plots and **b** p(r) curves of the *Pf*SRP9/14 heterodimer (green), *Pf*Alu118 RNA (magenta), and *Pf*Alu76 RNA (blue) are presented. **c** Ab initio model of *Pf*SRP9/14 superposed with the crystal structure of *Hs*SRP9/14 (PDB: 4UYK)^27^. The position of loop L1 is marked. **d**, **e** show ab initio models of *Pf*Alu118 and *Pf*Alu76 RNA, respectively.

Having assigned the secondary structure of the *Pf*Alu118 RNA by NMR, we aimed to obtain information on the 3D-architecture of the *Pf*Alu118 RNA and *Pf*SRP9/14 proteins. We first applied SAXS to the *Pf*SRP9/14 heterodimer and *Pf*Alu118 RNA (Supplementary Fig. 5) and calculated Kratky plots to characterize the degree of folding (Fig. 4a). For the *Pf*SRP9/14 heterodimer, the plot shows a bell-shaped curve characteristic of folded globular molecules. Consequently, the pairwise distance distribution (p(r)) function, describing the paired set of distances between all the electrons in the macromolecular structure, shows a compact conformation of the heterodimer with a *D*_max_ of 7.2 nm (Fig. 4b and Supplementary Table 2). Ab initio shape determination of the *Pf*SRP9/14 heterodimer using dummy atom modeling with DAMMIN/F^34,35^ shows that the envelope accommodates the crystal structure of the human SRP9/14 heterodimer (PDB: 4UYK)^27^ (Fig. 4c). A sequence alignment of SRP14 from different species reveals the presence of an extended (residues 35–70) internal loop L1 in *Pf*SRP14 (Supplementary Fig. 6). The additional density in the dummy atom model of *Pf*SRP9/14 that remains unoccupied by the crystal structure of *Hs*SRP9/14 may represent this loop, which is not visible in the published structures of the *Hs*SRP9/14 heterodimer^27,28^ (Fig. 4c).

The Kratky plot of the SAXS data from *Pf*Alu118 RNA show a bi-modal distribution, which is characteristic of multiple domains (Fig. 4a). The pairwise distribution function shows that *Pf*Alu118 RNA adopts a rather extended conformation with a *D*_max_ of 11.8 nm (Fig. 4b and Supplementary Table 2). The existence of a bi-modal distribution in both Kratky plot and p(r) curves of *Pf*Alu118 RNA reflects the presence of separate domains in the molecule. This observation is further confirmed by ab initio shape reconstructions using dummy atom modeling with DAMMIN/F^34,35^, revealing an open Y-shaped conformation of *Pf*Alu118 (Fig. 4d).

From the SAXS data, it is evident that helices H3 and H4 do not form a loop–loop pseudoknot, however the similarity in the length of helices H3, H4, and H5 hinder their unambiguous assignment within the SAXS envelope. In order to tackle this problem, we used *Pf*Alu76 RNA, which completely lacks helix H5 (Supplementary Fig. 5). Kratky and p(r) curves for *Pf*Alu76 also show a bi-modal distribution, pointing towards an extended modular organization within the isolated 5′ domain (Fig. 4a, b). The RNA adopts a completely open conformation with a *D*_max_ = 11.5 nm (Fig. 4b, Supplementary Table 2) and no signs of pseudoknot formation (Fig. 4e). A comparison of the shape models of the two RNA variants (Fig. 4d, e) helps to confirm the overall position of the 5′ and 3′ domains i.e. the position of helix H5 relative to helices H3 and H4.

### NMR-SAXS-based modeling of *Pf*Alu118 RNA

**Fig. 5:**
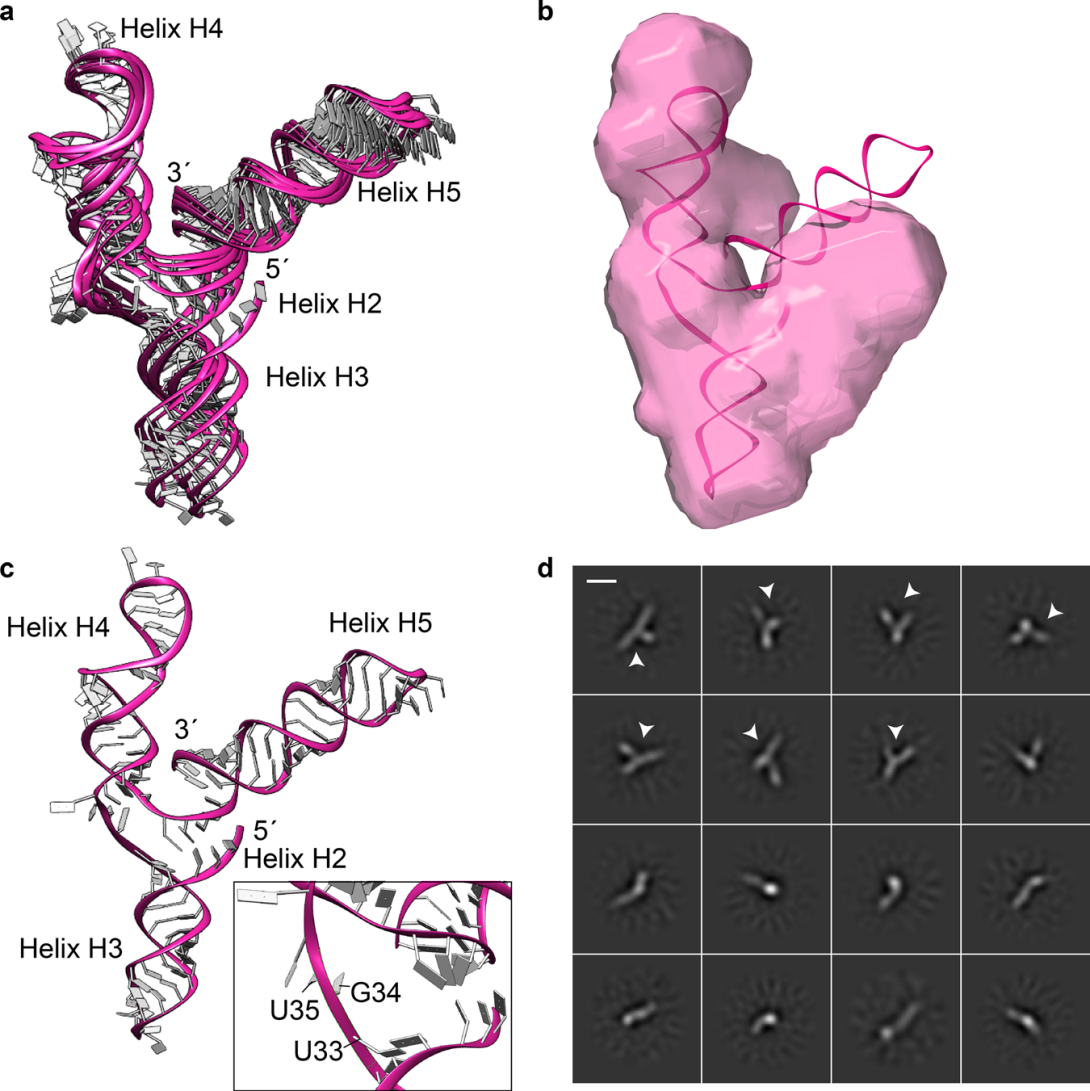
Structure analyses of the *Pf*Alu118 RNA. **a** NMR-SAXS-based atomic models of *Pf*Alu118 RNA. **b** Ab inito model of *Pf*Alu118 RNA superimposed with the top scored *Pf*Alu118 RNA NMR-SAXS model calculated by SREFLEX. **c** Top scored *Pf*Alu118 RNA NMR-SAXS model with inset showing the UGU motif exposed at the three-helix junction. **d** Single-particle cryo-EM analysis of *Pf*Alu118 RNA shown with representative reference-free 2D-class averages. Arrows mark class averages where the Y-shaped architecture of the RNA is visible. Scale bar represents 30 Å.

We next aimed to obtain an atomic model of the complete *Pf*Alu118 RNA using the experimental NMR and SAXS information. For this purpose, we used the FARFAR2 webserver^36^ with experimental NMR-based secondary structure restraints (Supplementary Fig. 7). A total of 500 decoy models were generated, ranked, and clustered to yield 10 low-energy cluster centers. Subsequently, they were scored according to their agreement with the experimental SAXS data and the best-scoring energy cluster (discrepancy *χ*^2^ = 4.18) was further subjected to normal mode analysis (NMA) with SREFLEX^37^. Here, a flexible refinement of the RNA model was performed in order to improve the overall agreement between the computed and experimental SAXS data. Using this approach, we obtained a final set of atomistic models of the *Pf*Alu118 RNA (Fig. 5a) yielding a significantly better agreement with the experimental SAXS data (*χ*^2^ ranging from 2.0 to 2.6) (Supplementary Fig. 7). All these models maintain the Y-shaped architecture of the RNA and their overall dimensions agree well with the low-resolution particle shape reconstructed ab initio (Fig. 5b). Systematic deviations observed between the NMA generated models and the ab initio shape and also minor misfits in Supplementary Fig. 7 (*χ*^2^ values exceeding unity) are likely to stem from the flexibility of *Pf*Alu118 RNA in solution, especially between its 5′ and 3′ domains. Of note, the coaxial stacking observed between helices H3 and H4 in the NMR-SAXS based atomic models is in agreement with the molecular envelope of the *Pf*Alu76 RNA (Fig. 4e). These models expose the UGU motif at the three-helix junction, which would be available for binding to the *Pf*SRP9/14 heterodimer in a canonical manner^27,28^ (Fig. 5c).

In a complementary approach to validate the overall Y-shaped architecture of the *Pf*Alu118 RNA, we used single-particle cryo-EM. The high contrast for RNA molecules arising from their phosphorus-rich backbone makes the study of small ~30–40 kDa RNAs possible^38,39^. However, the inherent conformational flexibility of RNAs limits the resolution of cryo-EM reconstructions. Here, we performed single-particle cryo-EM studies of *Pf*Alu118 RNA using a Glacios 200 kV microscope equipped with Falcon3 detector (Supplementary Fig. 8, Supplementary Table 3). The RNA particles showed a good distribution on raw micrographs (Supplementary Fig. 8a). Reference-free 2D classification clearly shows class averages with a Y-shaped conformation for *Pf*Alu118 RNA (Fig. 5d). Finer details of the RNA secondary structure are not visible, owing to its small size (~38 kDa) and inherent flexibility in the RNA. In addition, the near-equal lengths of the three arms of the RNA pose problems for unambiguous particle alignment during classification. Nevertheless, these data independently show that the *Pf*Alu118 RNA adopts an open Y-shaped conformation in agreement with the NMR-SAXS based atomic model and that a loop–loop pseudoknot is not formed between helices H3 and H4.

### Binding of the *Pf*SRP9/14 heterodimer does not induce closure of the *Pf*Alu domain

**Fig. 6:**
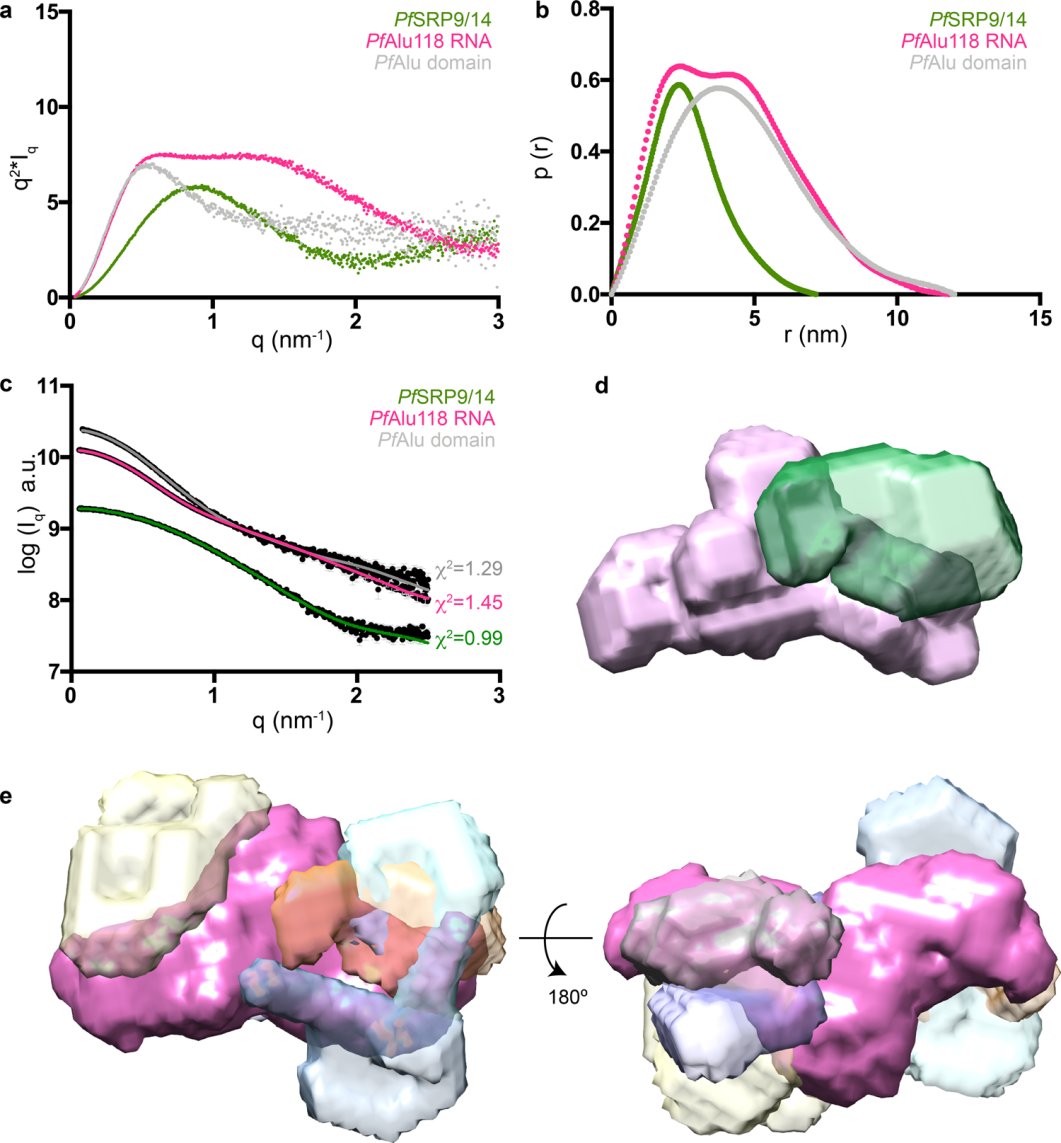
SAXS analysis of the *Pf*Alu domain. **a** Kratky plots and **b** p(r) curves of the *Pf*SRP9/14 heterodimer (green), *Pf*Alu118 RNA (magenta), and *Pf*Alu domain (gray) are presented. Data for *Pf*SRP9/14 heterodimer and *Pf*Alu118 RNA have been replicated from Fig. 4a, b. **c** MONSA modeling of *Pf*Alu domain. Out of 10 MONSA runs, the data for the top-scoring run are shown. **d** Multi-phase ab initio models corresponding to the best-scoring MONSA run in **c** are shown. The RNA phase is colored in pink and protein phase in green. **e** Aligned, averaged, and filtered ab initio models of the RNA phase from 10 independent MONSA runs are shown in magenta, whereas the different positions of the protein phase occupied on the RNA are shown in various light colors. The protein phases of individual runs do not show spatial convergence but are placed overall near the three-helix junction. a.u. represents arbitrary units.

In order to investigate if binding of the *Pf*SRP9/14 heterodimer results in a closed conformation of the RNA as observed in canonical Alu RNA structures^12,26–28^, SAXS analysis of the complete Alu domain was performed (Supplementary Fig. 9). The effect of *Pf*SRP9/14-binding to *Pf*Alu118 RNA was qualitatively assessed by the Kratky plot (Fig. 6a). Although free *Pf*Alu118 RNA reveals a Kratky plot that is characteristic for two globular domains, the *Pf*Alu domain has a bell-shaped curve at low *q* values, indicative of a less-flexible particle (Fig. 6a). The pairwise distribution function shows that the maximum dimension of the *Pf*Alu domain (*D*_max_ = 12 nm) does not change significantly in comparison with that of the free RNA (Fig. 6b, Supplementary Table 2). In order to probe the structure of the *Pf*Alu domain, we used multi-phase ab initio modeling (MONSA), which involves simultaneous fitting of experimental SAXS data for individual components (phases) together with those for the complex^40^. Simultaneous fitting of SAXS curves for *Pf*SRP9/14, *Pf*Alu118, and their complex shows good agreement between the obtained multi-phase models and the experimental data, as signified by the low *χ*^2^-fitting values (Supplementary Table 4). Improvements in the *χ*^2^ fitting value were not obtained when using only the individual RNA or protein components as constraints (Supplementary Table 4). This suggests that the overall conformation of *Pf*Alu118 RNA is not drastically altered upon binding to *Pf*SRP9/14 and it still maintains the open Y-shaped conformation. Multiple rounds of MONSA modeling were made and the best-scoring models show that the *Pf*SRP9/14 heterodimer is placed near the three-helix junction of the RNA (Fig. 6c, d). However, an alignment of MONSA models with respect to the RNA phase did not lead to consistent placement of *Pf*SRP9/14 between multiple runs (Fig. 6e). This could be attributed to the similar size of helices H3, H4, and H5, leading to the placement of *Pf*SRP9/14 on either side of the central arm of *Pf*Alu118 RNA.

Taken together, binding of *Pf*SRP9/14 to *Pf*Alu118 RNA does have a stabilizing effect as inferred from the Kratky plot analysis, but the RNA maintains the open Y-shaped conformation both with and without the *Pf*SRP9/14 heterodimer. Notably, the presence of a pseudoknot-closed conformation of the SRP Alu RNA is a feature conserved among all the previously deposited structures of the SRP Alu domain from a variety of species. Therefore, the open conformation without pseudoknot adopted by the *Pf*Alu domain is so far unique and raises questions about its binding mode at the ribosome.

### Ribosome binding of the *Pf*Alu domain

In order to analyze whether the open Y-shaped conformation of the Alu domain can be recognized by the ribosome, we used MST for the determination of binding affinities. For this purpose, we isolated non-translating empty 80 S ribosomes from *P. falciparum*-infected human erythrocyte cultures to homogeneity with slight modifications to previously published protocols^21,41–43^. We employed puromycin treatment to remove any ribosome-bound cofactors and used negative-stain electron microscopy as quality control to check homogeneity of *Pf*80S ribosome preparations. For MST, the *Pf*80S ribosomes were labeled using *N*-hydroxysuccinimide-ester (NHS-ester) dye, and thermophoresis was measured at concentrations of 5–20 nM.

**Fig. 7:**
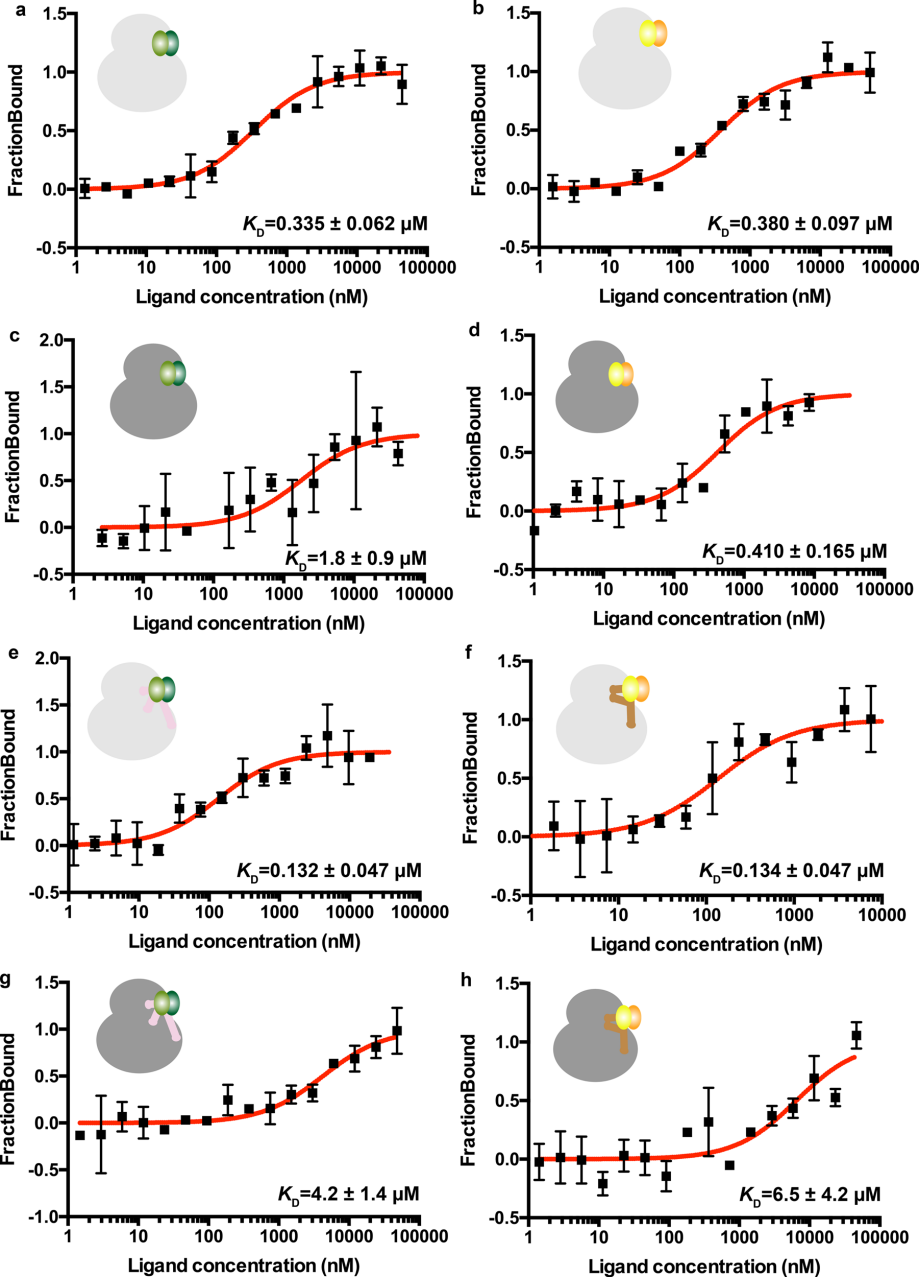
MST data for SRP9/14 and Alu domain binding to 80S ribosomes. *Pf*SRP9/14 and *Hs*SRP9/14 heterodimers are shown in light–dark green and yellow–orange, respectively. *Pf*80S ribosomes are shown in light gray, whereas *Hs*80S ribosomes are shown in dark gray. **a** The *Pf*SRP9/14 heterodimer binds to *Pf*80S ribosomes with high affinity. **b** *Hs*SRP9/14 binds to *Pf*80S ribosomes with slightly lower affinity. **c**, **d** *Pf*SRP9/14 binds to *Hs*80S ribosomes with substantially lower affinity than *Hs*SRP9/14. **e** *Pf*Alu or **f** *Hs*Alu domains bind to *Pf*80S ribosomes with similar high affinity. **g** *Pf*Alu or **h** *Hs*Alu domains bind to the *Hs*80S ribosomes with ~30- to 50-fold weaker affinity compared to *Pf*80S ribosomes (**e**, **f**). Average values for at least two replicates are shown and error bars represent the standard deviation (*n* ≥ 2).

First, MST measurements of *Pf*SRP9/14 heterodimer binding to *Pf*80S ribosomes revealed a dissociation constant *K*_D_ = 0.335 µM (Fig. 7a). Next, we wanted to compare if *Pf*80S ribosomes can also recognize human SRP9/14 proteins. We titrated the *Hs*SRP9/14 heterodimer with *Pf*80S ribosomes and measured only a slight decrease in binding affinity (*K*_D_ = 0.380 µM) (Fig. 7b). This is surprising as sequence comparisons of SRP9/14 from different eukaryotes show that a subset of residues indispensable for SRP-mediated elongation arrest activity^16,44^ is either mutated (K70 in helix α2 of SRP9 has a charge reversal mutation) or completely absent (C-terminal tail of SRP14 containing positively charged residues) in *Pf*SRP9/14 (Supplementary Fig. 6). Therefore, to understand if these differences between *Pf*SRP9/14 and *Hs*SRP9/14 proteins lead to differential recognition by the human 80S ribosomes, we repeated the MST measurements using non-translating empty *Hs*80S ribosomes purified from HeLa cells. Although *Pf*SRP9/14 was found to bind to *Hs*80S ribosomes with a ~4.5-fold weaker affinity (*K*_D_ = 1.8 µM), the *Hs*SRP9/14 heterodimer bound with a comparable affinity (*K*_D_ = 0.410 µM) as to *Pf*80S ribosomes (Fig. 7c, d).

We next tested whether the open Y-shaped conformation of the *Pf*Alu domain could be accommodated by the *Pf*80S ribosome. We observed that the complete *Pf*Alu domain binds to *Pf*80S ribosomes 2.5-fold stronger than the heterodimer alone with a low nanomolar affinity (*K*_D_ = 0.132 µM) (Fig. 7e). In order to analyze whether a pseudoknot-closed conformation of the Alu domain is also recognized by the *Pf*80S ribosome, we reconstituted the *Hs*Alu domain using an Alu RNA construct^28^ corresponding to our *Pf*Alu118 RNA (Supplementary Fig. 3a). Remarkably, the closed *Hs*Alu domain binds to *Pf*80S ribosomes with a similarly high affinity (*K*_D_ = 0.134 µM) (Fig. 7f). In stark contrast, the *Hs*80S ribosome is unable to provide a high-affinity binding site for either the *Pf*Alu or *Hs*Alu domain (micromolar affinities) (Fig. 7g, h).

Taken together, our data show that the open Y-shaped *Pf*Alu domain binds to *Pf*80S ribosomes with high affinity. The ~2.5-fold increase in binding affinity of the complete Alu domain for *Pf*80S ribosomes compared with the SRP9/14 heterodimer alone highlights the affinity contribution of the Alu RNA in both homologous and heterologous systems.

## Discussion

The SRP Alu domain has been reported to confer translation elongation arrest to ribosomes in eukaryotes^16,17^ and prokaryotes^14^. Translation arrest or retardation is thought to provide SRP enough time to properly engage with the membrane-associated translocon^18^. Therefore, such translation retardation might be beneficial in eukaryotes owing to compartmentalization and longer targeting distances^14^ or even in certain prokaryotes during specific growth stages such as sporulation, where extremely efficient targeting is required^45^. Prokaryotic and mammalian SRP are structurally and functionally well-characterized, but our understanding of lower eukaryotes is limited. Remarkable variations exist in the composition of the Alu domain, which has been attributed to its rapid evolution^8,46^. Here, we have characterized the structure of SRP Alu domain from the malarial parasite *P. falciparum*, which harbors modifications in its Alu RNA, and studied its ribosome-binding properties.

Using a short Alu RNA construct only comprising the 5′ domain, we show that helices H3 and H4 maintain an open conformation without forming the loop–loop pseudoknot conserved in canonical Alu folds. In presence of the 3′ domain (helix H5), the RNA still adopts an open Y-shaped conformation, suggesting an absence of the tertiary interactions between the 5′ and 3′ domains induced by the jack-knife closure of canonical Alu domains. In contrast to the *Hs*Alu RNA, which adopts a strictly closed-pseudoknot conformation^28^, this open conformation of *Pf*Alu RNA remains unaltered upon binding of the SRP9/14 proteins. The conserved binding site for SRP9/14 on the Alu RNA is the UGU motif that forms an RNA U-turn^28^. Interestingly, in the bacterial SRP Alu where the SRP9/14 heterodimer is absent, the UGU motif itself does not form a U-turn and the closure of helices H3 and H4 is achieved by the formation of an extra intra-strand base pair^26^. In the case of the *Pf*Alu domain, the closure of helices H3 and H4 occurs neither in the absence nor the presence of *Pf*SRP9/14, despite the Kratky plot analysis indicating that binding of SRP9/14 stabilizes the RNA. It is likely that the long insertion in *Pf*SRP14 loop L1 (Supplementary Fig. 6) binds near the three-helix junction of *Pf*Alu RNA and therefore stabilizes the overall fold of the RNA. Of note, in bacterial and archaeal SRP Alu domains, rigidity at the three-helix junction in the absence of SRP9/14 is provided by an additional RNA helix H1^26,27^.

**Fig. 8:**
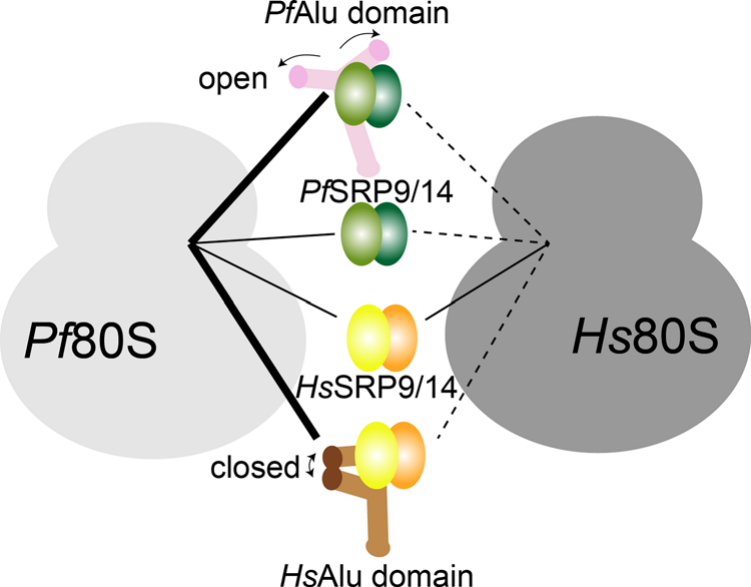
Model of SRP Alu domain–ribosome interactions. Schematic diagram outlining the *Pf* and *Hs*Alu domain–ribosome interactions. Dashed lines indicate weak µM binding, thin solid lines weak nM binding, and thick solid lines indicate strong nM binding. The open and closed conformations of the Alu domain are marked.

Using MST-binding studies, we show that the *Pf*SRP9/14 heterodimer can bind to non-translating *Pf*80S ribosomes with high affinity even in the absence of Alu RNA. This is not surprising, as cryo-EM reconstructions of mammalian SRP bound to RNCs show a direct interaction of the proteins with 18S rRNA at the FBS within the 40 S subunit^6,14,20^. Of note, the positively charged C-terminus of SRP14, which is essential for elongation arrest activity^17^ and binding to 40S subunits during stress granule formation in humans^47^, is absent in *P. falciparum* (Supplementary Fig. 6). Nevertheless, the *Pf*SRP9/14 heterodimer still binds to cytosolic *Pf*80S ribosomes with similar affinity, as is also the case for *Hs*SRP9/14. It is therefore puzzling that the *Pf*SRP9/14 heterodimer binds to *Hs*80S ribosomes with a ~4.5-fold weaker affinity compared to *Hs*SRP9/14 (1.8 µM compared with 0.410 µM, Fig. 7c, d). These data suggest a distinct mode of SRP9/14 protein interaction with ribosomes from humans and the malarial parasites. Our MST experiments also show that while *Pf*80S ribosomes are able to recognize and bind both open and closed conformations of the Alu domain from different species, *Hs*80S ribosomes show very weak binding to isolated Alu domains (Fig. 8). These data are indicative of either plasticity of the *Pf*80S ribosome, enabling the accommodation of both the open and closed conformations of the Alu domain with equal affinity, or the binding to different ribosomal sites.

Intriguingly, a detailed comparison of SRP RNAs also reveals that the linker helix H5 connecting the 5′ Alu and the 3′ S domain of the SRP RNA significantly differs in length between the two species, with *Pf*SRP RNA being ~20 base pairs shorter. In the context of full-length SRP, where the S domain associates at the ribosomal tunnel exit and the Alu domain binds at the FBS, the shortened helix H5 in *Pf*SRP RNA might pose an additional strain on the SRP particle and limit the flexibility of the two domains on the ribosome. It is also possible that owing to a shorter RNA, the Alu domain cannot reach its canonical binding site at the FBS on the ribosome.

In order to further understand the difference between the parasitic and human ribosomes, we compared the FBS (L7/L12 stalk of the 60S subunit) where the SRP Alu domain is known to bind. In recent cryo-EM structures of the *P. falciparum* ribosome bound to an anti-malarial drug^43^ or tRNAs^48^, the subunit interface proteins uL10 and uL11 at the L7/L12 stalk were not modeled owing to considerable flexibility. This is consistent with our own attempt to obtain a cryo-EM reconstruction of *Pf*80S in complex with the *Pf*Alu domain. Despite the high-affinity binding of the *Pf*Alu domain to *Pf*80S, we could not observe additional density corresponding to the Alu domain on the ribosome. The L7/L12 stalk is not sufficiently well ordered even in the cryo-EM structure of the mammalian SRP-RNC complex in the engaged state^20^, making it impossible to make a detailed model of the interactions. Nevertheless, structural adaptations in the binding mode of SRP to ribosomes have been reported in other species. For example, in *B. subtilis* the absence of interactions with the 40S subunit is compensated for by additional interactions with rRNA helices at the base of the L7/L12 stalk^14^, resulting in a functional SRP Alu domain capable of elongation arrest^14^.

We have previously shown that the SRP S domain forms a high-affinity interaction with both RNCs and empty human 80 S ribosomes (*K*_D_ < 5 nM) and that the Alu domain is not a primary ribosome-binding determinant^21^. Here, we complete these data and reveal that although the *Hs*SRP9/14 heterodimer binds to empty ribosomes with nanomolar affinity, the complete Alu domain indeed binds much more weakly with a *K*_D_ ~6.5 μM possibly owing to charge repulsions as observed for the entire *Hs*SRP RNA^21^. In line with these observations, it has been reported that the mammalian SRP can stably engage with RNCs, whereas the Alu domain is still detached^20^. The specific and stable engagement of the SRP S domain with RNCs is thought to provide a kinetic advantage to the Alu domain, which could be recruited to the FBS owing to an effective increase in its local concentration once SRP is engaged with the nascent chain^20^. It is therefore intriguing that *Pf*80S ribosomes can associate with isolated Alu domains with high nanomolar affinity. We suggest that, in contrast to the two-step binding model suggested for the mammalian system^20^, simultaneous high-affinity binding of the SRP S and Alu domains upon recruitment to the RNCs could provide an advantage for successful protein targeting. In the engaged state of SRP with mammalian RNCs, the SRP S domain is tightly associated due to its interactions with both the tunnel exit and the nascent chain, whereas elongation factors could displace the Alu domain from the FBS. The competition between elongation factors eEF1 and eEF2 and the Alu domain could then contribute towards the observed translation retardation by SRP^20,49^. Higher affinity binding of the SRP Alu domain could confer better displacement of the elongation factor, possibly increasing the time window for the successful delivery of the protein cargo. This would mean that binding of the Alu domain to parasitic ribosomes has been adapted to improve the overall accuracy of co-translational protein targeting. Further studies will be required to determine whether the SRP Alu domain has an effect on elongation arrest and protein targeting efficiency in *P. falciparum*. However, testing for elongation arrest activity in a homologous cell-free translation system derived from *P. falciparum* is challenging, as is the reconstitution of complete *Pf*SRP. Nevertheless, several studies have provided evidence for increased fidelity of parasitic ribosomes, which possess an altered exit tunnel to accommodate poly-lysine repeats important in host-cell adhesion and invasion^50^ and have adapted to efficiently translate its AT-rich (81%) genome^50–52^. Importantly, chimeric yeast ribosomes containing parasitic rRNA that harbors differences at the FBS (Supplementary Fig. 1) displayed increased translation accuracy^53^. Therefore, in light of ribosome-profiling experiments that show a tremendous increase in protein synthesis during the late blood stages of *P. falciparum*^51,52^, it is plausible that the SRP Alu domain–ribosome interactions have evolved in *P. falciparum* to form a more promiscuous and efficient protein targeting machinery. This might be essential for parasite survival considering the vast amount of protein it needs to secrete to successfully complete its life cycle.

In summary, we have shown that the SRP Alu domain of *P. falciparum* adopts an open Y-shaped conformation without the consensus loop–loop pseudoknot. This is a feature of the Alu domain, which has so far not been reported for prokaryotes or eukaryotes. Although the open-parasitic and closed-human Alu domains only weakly associated with the human ribosome, the parasitic ribosome can accommodate both with nanomolar affinity. Our study extends the current understanding of species-specific differences and alterations in SRP Alu domain–ribosome interactions and sets the path for detailed future studies.

## Methods

### Cloning

The coding sequences for SRP9 and SRP14 from *P. falciparum* with codon optimization for *E. coli* expression were obtained by gene synthesis (MWG Biotech AG, part of Eurofins Genomics, Germany, Luxemburg). *Pf*SRP9 (residues 1–103) and *Pf*SRP14 (residues 1–104) were cloned into pET24d and modified pET16b vector containing N-terminal His-Maltose binding protein tag upstream of Tobacco Etch Virus (TEV) protease cleavage site using *Nco*I/*Bam*HI restriction enzymes, respectively. Similarly, *Hs*SRP9 (residues 2–86) and C-terminally truncated *Hs*SRP14 (residues 1–107) were cloned into pET24d and modified pET16b, respectively.

The DNA sequences encoding the corresponding SRP Alu RNAs from *P. falciparum* (Genbank HG323585.1) were generated by performing overlapping PCR of complementary oligonucleotide sequences. Sequences encoding *Pf*Alu76 (nts 4–73) and *Pf*Alu118 (nts 3–94 and 279–298) were cloned into pUC19 vector with *Eco*RI/*Hin*dIII restriction sites. Self-cleaving hammerhead ribozyme was fused at the 3′-ends of RNA constructs to obtain uniform ends. Individual stem-loop helices H3 (nts 5–33), H4 (nts 38–72), and H5 (nts 77–94 and 279–298) were purified for NMR experiments. Similarly, sequence encoding *Hs*Alu RNA (Genbank X04248.1) (nts 3–64 and 283–298), fused to hammerhead ribozyme at the 3′-end was cloned into pUC19 vector using *Eco*RI/*Hin*dIII restriction sites. In all RNA constructs, the S domain was replaced by a GUAA tetraloop. Specific point mutations at the 5′- and 3′ ends of constructs were introduced for enhancement of T7 polymerase activity and hammerhead cleavage efficiency (see Supplementary Fig. 3 for sequence details of all RNA constructs used in this study).

### Proteins/RNA purification

For purification of *Pf*SRP9/14 or *Hs*SRP9/14 heterodimers, the respective plasmids were co-transformed in BL21 (DE3) Rosetta2 electrocompetent cells, grown at 37°C up to an OD of 1 in auto-induction media^54^ and subsequently co-expressed at 23°C for ~16 hr. Cells were lysed in buffer containing 50 mM Tris pH 7.5, 200 mM NaCl, 10 mM MgCl_2_, 10 mM KCl, 20 mM imidazole, and 2 mM β-mercaptoethanol. The heterodimer was purified over a 5 ml His-Trap FF column (GE Healthcare) with elution buffer containing 300 mM imidazole. Next, overnight tag-cleavage using TEV protease and simultaneous dialysis of the proteins into 20 mM HEPES pH 7.5, 150 mM NaCl, 10 mM MgCl_2_, 10 mM KCl, 1 mM DTT was carried out. The heterodimer was further purified using a 1 ml Resource S cation exchange column (GE Healthcare), where the complex was eluted with a linear gradient of 150 mM NaCl to 1 M NaCl. Finally, the respective heterodimer was polished using gel filtration (Superdex 75 16/60) column (GE Healthcare) equilibrated with SEC buffer containing 20 mM HEPES pH 7.5, 150 mM NaCl, 10 mM MgCl_2_, 10 mM KCl, and 1 mM DTT. *Pf* and *Hs*Alu RNAs were produced by in vitro transcription using T7 polymerase as described earlier^55^. The RNAs were purified by denaturing polyacrylamide gel electrophoresis and finally desalted into water using a PD-10 desalting column (GE Healthcare). For in vitro reconstitution of the protein–RNA complexes, the RNA was first refolded using a snap-cool protocol. The RNA was heated to 95°C for 5 min, snap cooled on ice for 5 min, diluted with 10× refolding buffer containing 200 mM HEPES pH 7.5, 1.5 M NaCl, 100 mM MgCl_2_, and 100 mM KCl and incubated at 37 °C for 10 min. Subsequently, the *Pf*/*Hs*SRP9/14 heterodimer was added in 1.2-fold excess and purified over the size-exclusion column.

### Purification of *Pf*80S and *Hs*80S ribosomes

*Pf*80S ribosomes were isolated from wild-type NF54 strain of *P. falciparum*-infected human RBCs (blood group O) maintained at a hematocrit of ~3.5% and 3–5% parasitemia. Parasites were grown in 100 mL human blood suspension cultures (10^10^ RBCs/mL blood) in RPMI-1640 buffer with 25 mM HEPES pH 7.3, 0.2 mM hypoxanthin, 12.5 µg/mL gentamycin (Roth) supplemented with 0.5% albumax. Synchronization of the parasites was done by sorbitol treatment. Finally, RBCs were lysed (at the Schizont stage of the parasites) using 0.15% saponin to obtain ~1.5 × 10^9^ parasites/mL of blood. After rigorous washing with PBS, the parasites were flash-frozen at −80 °C until further use. To obtain *Pf*80S ribosomes a previously published protocol with slight modifications was employed^21^. Parasites obtained from 4 mL blood cultures were resuspended in 10 mL buffer containing 50 mM HEPES pH 7.5, 300 mM KOAc, 6 mM Mg(OAc)_2_, 0.5% (v/v) NP40 (Sigma-Aldrich), RNAsin plus (Promega), protease inhibitor (Sigma-Aldrich), and 1 mM TCEP for 30 min on ice and the lysate was cleared at 20,000 × *g* for 15 min. The supernatant was loaded on a 5 mL 30% (w/v) sucrose cushion prepared in cushion buffer containing 20 mM HEPES pH 7.5, 150 mM KOAc, 2 mM Mg(OAc)_2_, RNAsin plus (Promega), protease inhibitor (Sigma-Aldrich), and 1 mM TCEP and centrifuged at 116,000 × *g* for 16 h in a T-865 rotor (Thermo Fisher). The pellet was carefully resuspended on ice for 3 h in 500 μL buffer containing 20 mM HEPES pH 7.5, 150 mM KOAc, 6 mM Mg(OAc)_2_, 6.8% (w/v) sucrose, RNAsin plus (Promega), protease inhibitor (Sigma-Aldrich) and 1 mM TCEP. 80 S monosomes were generated by the addition of 1 mM puromycin (Thermo Fisher) and incubation for 15 min at 37°C and 1 h on ice with intermittent mixing. The monosomes were loaded onto 24 mL of a 15–40% (w/v) sucrose gradient prepared in cushion buffer. The samples were centrifuged at 60,000 × *g* for 17 h in a Surespin 630 rotor (Sorvall). The gradient was harvested manually from top to bottom in 1 mL fractions and the absorbance measured at 260 nm (1 OD260 = 84 μg, 20 nM). The monosome peak was collected and concentrated in a centrifugal filter device (100 kDa MWCO, Millipore), diluted 10× in buffer containing 20 mM HEPES pH 7.5, 100 mM KOAc, 5 mM Mg(OAc)_2_, and 1 mM TCEP to reach a concentration of <3% (w/v) sucrose and concentrated again to ~1 mg/mL, flash-frozen in 25 μL aliquots and stored at −80°C until further use. *Hs*80S ribosomes were isolated from HeLa cells essentially as described before^21^.

### Electrophoretic mobility shift assay

Protein–RNA complex formation between *Pf*Alu118 RNA and *Pf*SRP9/14 heterodimer and between *Hs*Alu RNA and *Hs*SRP9/14 heterodimer was monitored by using agarose gel electrophoresis. In all, 10 µL reactions containing a 0, 0.5, one-, two, and threefold molar excess of the proteins over 4 µM RNA were incubated in SEC buffer for 30 min at room temperature (RT). The samples were supplemented with 10% (v/v) glycerol and run on Tris-borate agarose gels using 0.5× Tris-borate buffer at 80 V for 45 min at 4°C and subsequently imaged using ethidium bromide.

### Multi-angle light scattering

A total of 100 μL of the *Pf*Alu118 RNA, *Pf*SRP9/14 heterodimer and *Pf*SRP Alu complex at ~1 mg/mL were subjected to SEC using a Superdex 200 10/300 column (GE Healthcare) coupled to a MALS system (miniDAWN Tristar, Wyatt Technologies) and refractive index detector (RI-71, Shodex) at 4°C. The SEC column was pre-equilibrated with buffer containing 20 mM HEPES pH 7.5, 150 mM NaCl, 10 mM MgCl_2_, 10 mM KCl and 1 mM DTT, and the data were analyzed using Astra 6 software (Wyatt Technology).

### NMR spectroscopy

All spectra were recorded at 277 K on Avance III Bruker NMR spectrometer with proton Larmor frequency of 700 or 800 MHz, equipped with RT or cryogenic triple resonance gradient probes, respectively, in a buffer containing 20 mM sodium phosphate pH 6.8. *Pf*Alu RNA samples for helices H3, H4, and H5, were prepared by refolding in water and subsequent dilution in NMR buffer to obtain a final RNA concentration of ~250–300 µM. The samples were supplemented with 10% D2O. ^1^H, ^1^H-NOESY spectra were recorded with 200 ms mixing time, processed with NMRPipe^56^, and analyzed using CCPNmr analysis^57^.

### Small-angle X-ray scattering

All SAXS measurements were performed at 20°C using BioSAXS beamline BM29 with a 2D Pilatus detector at the European Synchrotron Radiation Facility (ESRF), Grenoble. Ten frames (for *Pf*SRP9/14) or 20 frames (for *Pf*Alu76, *Pf*Alu106, *Pf*Alu118 RNAs) with 0.5 s/frame exposure time were recorded using an X-ray wavelength of *λ* = 0.9919 Å in flow mode. For the *Pf*Alu domain protein–RNA complex, a gel filtration column (Superdex 200 10/300) was coupled to the SAXS measurement, where the sample was injected on the column with data being recorded continuously with an exposure of 1 sec/frame. The dedicated beamline software BsxCuBE was used for data collection and initial processing. 1D scattering intensities of samples and buffers were expressed as a function of the modulus of the scattering vector *q* = (4π/*λ*)sinθ with 2θ being the scattering angle and *λ* the X-ray wavelength. Downstream processing after buffer subtraction was done with PRIMUS^58^. Size-exclusion coupled SAXS data were viewed and processed with CHROMIXS^59^. *R*_g_ was determined using Guinier approximation and from p(*r*) curves. Dummy atom models for *Pf*SRP9/14, *Pf*Alu76 RNA, and *Pf*Alu118 RNA were generated using twenty DAMMIF^34^ runs, averaged and filtered using DAMAVER^60^, and finally refined with DAMMIN^35^. For MONSA^35^, SAXS profiles of *Pf*SRP9/14 heterodimer, free RNA, and complex were fitted simultaneously. For the protein and RNA, the ratio of 1:2 was used, their volumes were estimated using the volume approximations *V* = (*M*_W_/1.35) × 1.66 and (*M*_W_/1.74)*1.66, respectively. Ten independent MONSA runs were performed and the models were aligned and averaged using the RNA phase. Theoretical molecular weight estimates of the protein and RNA components were made using ProtParam^61^ and OligoCalc^62^ webservers, respectively.

### NMR-SAXS-based RNA modeling

NMR-based secondary structure restraints were provided as input to the RNA-modeling FARFAR2 webserver^36^. Non-canonical base pairs including U13–U25, U14–U24, and G95–A279 were provided as unpaired bases. To maintain continuity with helix H5 at the three-helix junction, the four bases in helix H2 were provided as paired. In total, 37 base-pair restraints were provided. Out of the 500 decoy models generated by FARFAR2, 400 lowest energy models were clustered using a cluster radius of 5 Å to produce 10 cluster centers, which were further scored using SAXS based *χ*^2^ fitting values with CRYSOL^63^. The top-scoring cluster was further refined using SREFLEX^37^. A detailed schematic of the modeling workflow is summarized in Supplementary Fig. 7.

### Cryo-electron microscopy

*Pf*Alu118 RNA was snap cooled in buffer containing 20 mM HEPES pH 7.5, 1 mM MgCl_2_ at a concentration of 1.6 mg/mL. In all, 3 μL of the sample was applied to Quantifoil R 2/1 holey carbon grids, which were glow discharged for 45 sec using PELCO easiGlow. The grids were blotted for 5 sec using a blot force of 10 at 100% humidity using Vitrobot Mark IV (FEI) operated at 4°C and immediately plunge-frozen in liquid ethane cooled with liquid nitrogen. A small cryo-EM data set was acquired on Glacios transmission electron microscope (Thermofischer) equipped with Falcon3 detector, operated at an acceleration voltage of 200 kV at an object pixel size of 1.56 Å. Micrographs were acquired using dose fractionation to record 32 frames per exposure with a dose rate of 1.99 e/Å^2^ per frame. Data were collected using EPU software package. All steps of image processing are summarized in Supplementary Fig. 8. Image processing was performed with RELION 3.1-beta software package^64^. Movie stacks were motion-corrected using MotionCor2 with 5 × 5 as the number of patches^65^ and estimation of contrast transfer function was performed with Gctf on the motion-corrected micrographs^66^. To generate templates for auto-picking, ~1400 particles were manually picked from 41 micrographs, subjected to unsupervised 2D classification. Classes depicting the RNA-like structure were used for auto-picking on all micrographs yielding 94291 particles. Particles were extracted using a box size of 164 × 164 pixels and subjected to 2D classification.

### Microscale thermophoresis

For MST measurements, *Pf*80S or *Hs*80S ribosomes were labeled with Atto-647 NHS-ester dye (*N*-HydroxySuccinimide, Thermo Fisher). The dye stock (1.15 mM in dimethyl sulfoxide) was diluted to a final concentration of ~30–45 μM in MST buffer containing 20 mM HEPES pH 7.5, 150 mM KOAc, 5 mM Mg(OAc)_2_, 1 mM TCEP, and ~100–200 nM ribosomes. The labeling reaction was incubated for 30 min at RT in the dark. The free dye molecules were removed by purification using a desalting spin-column (Zeba Spin 7 kDa cutoff, Thermo Fisher) equilibrated with MST buffer and the concentration of the labeled ribosomes was adjusted to 10–20 nM. The *Pf*SRP9/14 and *Hs*SRP9/14 heterodimers were also buffer exchanged to the MST buffer using the desalting columns. The protein–RNA complexes for *Pf*Alu and *Hs*Alu domains were prepared in SEC buffer by incubating 1.1-fold molar excess of proteins over the respective RNAs at RT for 30 min. In all, 0.5 mL (Amicon-Ultra) centrifugal filters with 30 kDa cutoff were used to remove excess proteins during simultaneous buffer exchange and concentration steps in the MST buffer. 16-serial dilutions of the ligand samples (*Pf* or *Hs* heterodimer/Alu domain complex) were mixed with labeled ribosomes in a 1:1 ratio and incubated at RT for 10 min before being loaded in premium coated glass capillaries (NanoTemper Technologies).

MST measurements were performed at 20°C using a Monolith NT.115 (NanoTemper Technologies) instrument with LED power ranging from 80 to 100% according to the input concentration of the fluorophore. Each measurement was typically done at three infrared-laser (MST) powers ranging from 20 to 70%. During each measurement, the initial fluorescence signal, MST, and final fluorescence signals were recorded for 5, 30, and 5 sec, respectively. Data were analyzed using the MO.Affinity Analysis software (NanoTemper Technologies) using default time windows. Data from two or three independent measurements (at the same MST power) were merged to obtain one data set for which averaged data points and error bars, representing standard deviation are shown. Data were fitted using a *K*_D_-model, which describes a molecular interaction with a 1:1 binding stoichiometry.

### Reporting summary

Further information on research design is available in the Nature Research Reporting Summary linked to this article.

## Supplementary information

Supplementary Information

Description of Additional Supplementary Files

Supplementary Data 1

Reporting Summary

## Data Availability

NMR imino resonance assignments have been deposited to the BMRB for *Pf*Alu RNA helices H3, H4, and H5 with accession codes: 50570, 50571, and 50572, respectively. SAXS data for *Pf*SRP9/14 heterodimer, *Pf*Alu118 RNA, *Pf*Alu76 RNA, *Pf*Alu118 RNA in complex with *Pf*SRP9/14 heterodimer (*Pf*Alu domain) have all been deposited to the SASBDB with accession codes: SASDK24, SASDK34, SASDK44, and SASDK54, respectively. All source data underlying the graphs are presented in Supplementary Data 1.
