# Supplementary material for: Structural analysis of the SRP Alu domain from Plasmodium falciparum reveals a non-canonical open conformation
Source: Commun Biol. 2021 May 20;4:600. doi: 10.1038/s42003-021-02132-y (PMC8137916; doi:10.1038/s42003-021-02132-y)
Supplement: Supplementary file 1 — Supplementary Information [file 42003_2021_2132_MOESM1_ESM.pdf]

SUPPLEMENTARY INFORMATION

Structural analysis of the SRP Alu domain from *Plasmodium falciparum* reveals a non-canonical open conformation

Komal Soni[1], Georg Kempf[1], Karen Manalastas-Cantos[2], Astrid Hendricks[1], Dirk Flemming[1], Julien Guizetti[3], Bernd Simon[4], Friedrich Frischknecht[3], Dmitri I. Svergun[2], Klemens Wild[1], Irmgard Sinning[1*]

Contents

Supplementary Figure 1 2
Supplementary Figure 2 3
Supplementary Figure 3 4
Supplementary Figure 4 5
Supplementary Figure 5 6
Supplementary Figure 6 6
Supplementary Figure 7 7
Supplementary Figure 8 8
Supplementary Figure 9 8
Supplementary Table 1 9
Supplementary Table 2 10
Supplementary Table 3 11
Supplementary Table 4 11
Supplementary References 12

Supplementary Figure 1

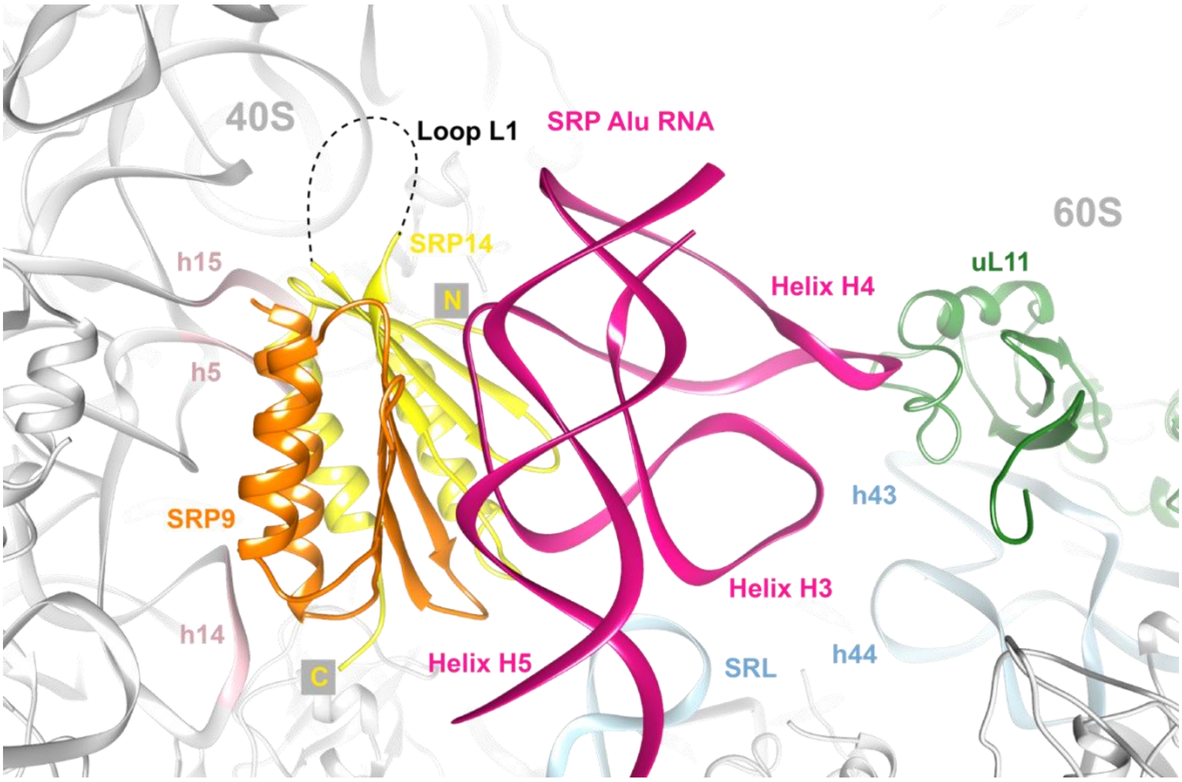

Supplementary Fig. 1: Cryo-EM reconstruction of a mammalian SRP-ribosome complex
Binding mode of a mammalian SRP Alu domain with its two interfaces to the 40S and 60S subunits shown[1] (PDB: 3JAJ). SRP Alu RNA, SRP9 and SRP14 are shown in magenta, orange and yellow respectively. SRP14 mainly contacts helices h5 and h15 of 18S rRNA, while SRP9 additionally contacts helix h14 (contacts shown in light pink). The N- and C-termini of SRP14 are marked and its flexible internal loop L1 is shown with dashes. 60S uL11 protein which interacts with SRP Alu RNA is shown in green. Additionally, helices h43, h44 and sarcin-ricin loop (SRL) of the 28S rRNA that are in close proximity to the Alu RNA are colored in light blue.

Supplementary Figure 2

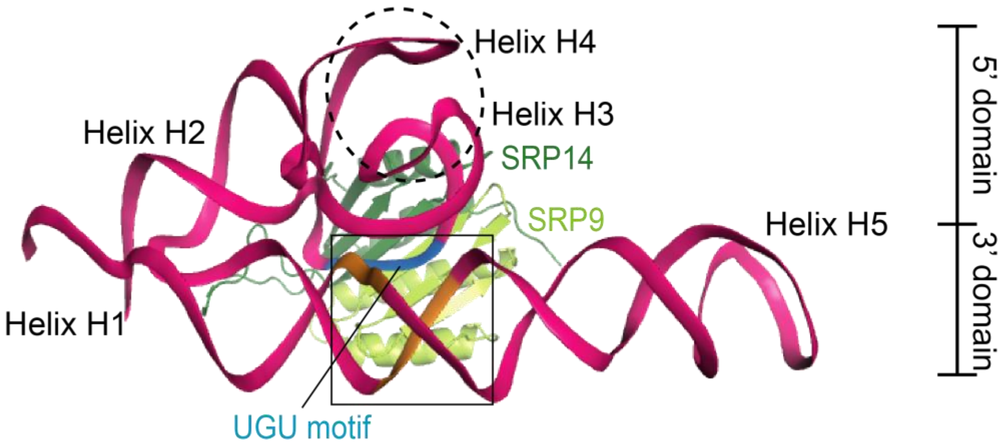

Supplementary Fig. 2: Structural features of canonical SRP Alu domains
Structural details of a chimeric SRP Alu domain with SRP9/14 proteins from *H. sapiens* and Alu RNA from *P. horikoshii*[2] shown, highlighting the overall conserved architecture of the domain (PDB: 4UYK). The UGU motif is indicated in blue and bases of the 3' domain involved in stabilizing contacts between 5' and 3' domains are shown in orange and boxed. The loop-loop pseudoknot between helices H3 and H4 is marked with a dashed circle.

Supplementary Figure 3

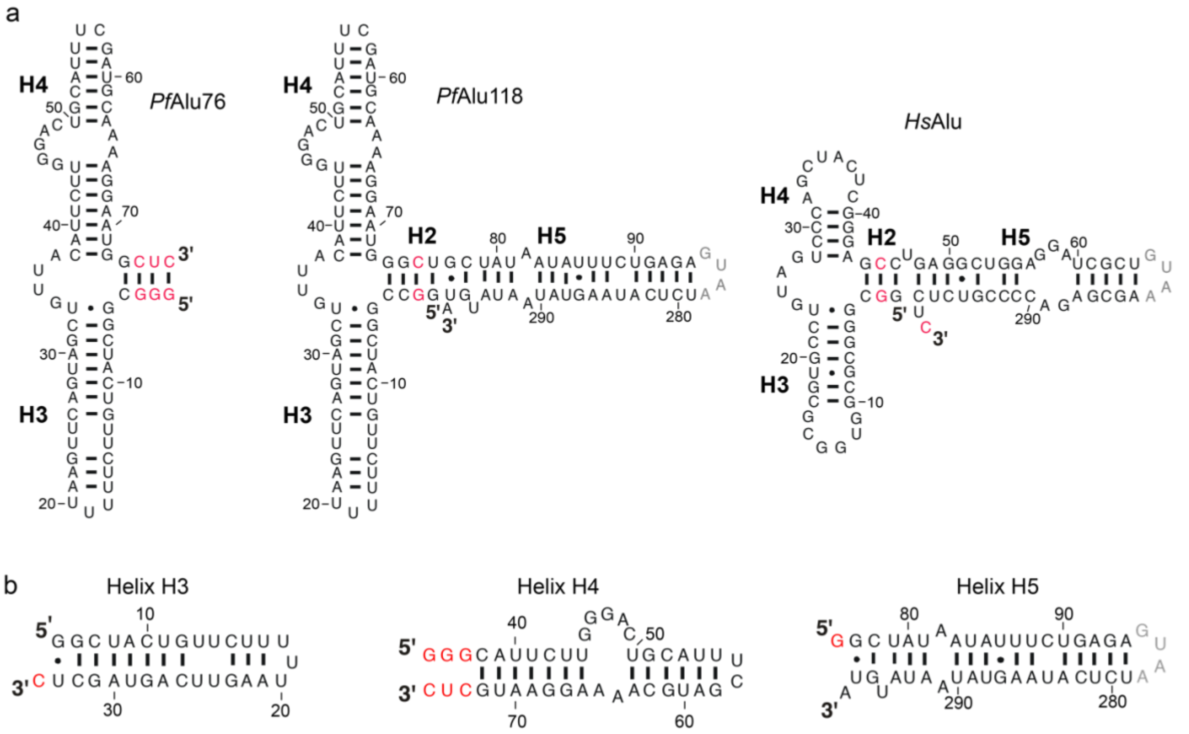

Supplementary Fig. 3: Constructs of *Pf*SRP Alu RNA
a 76 and 118 nucleotide RNA constructs from *Plasmodium falciparum* are shown on the left and the Alu RNA construct from *Homo sapiens* is presented on the right. **b** Individual helices H3, H4 and H5 derived from *Pf*Alu118 RNA for NMR studies are shown. Representative base pairing between the RNA bases as obtained from previous predictions[3] and SRPDB (https://rth.dk/resources/rnp/SRPDB/) is shown. Nucleotides colored in red are either point mutations inserted for 3'-Hammerhead ribozyme self-cleavage or for efficient transcription by T7 RNA polymerase. The S domain is always replaced by a GUAA tetraloop (colored in gray).

Supplementary Figure 4

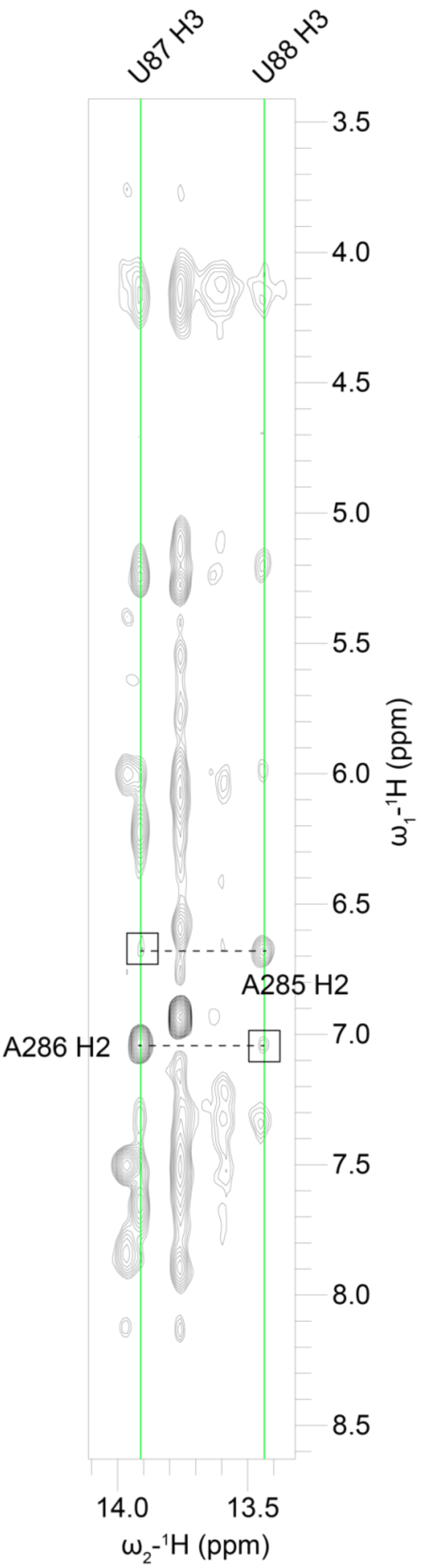

Supplementary Fig. 4: Assignment of helix H5 U88 imino proton
Zoom-in of the imino region of two dimensional ^{1}H,^{1}H-NOESY spectra of helix H5 with strips corresponding to U87 and U88 H3 imino protons marked in green. Cross-peaks between A286 H2 proton and U88 imino and A285 H2 proton and U87 imino are labeled.

Supplementary Figure 5

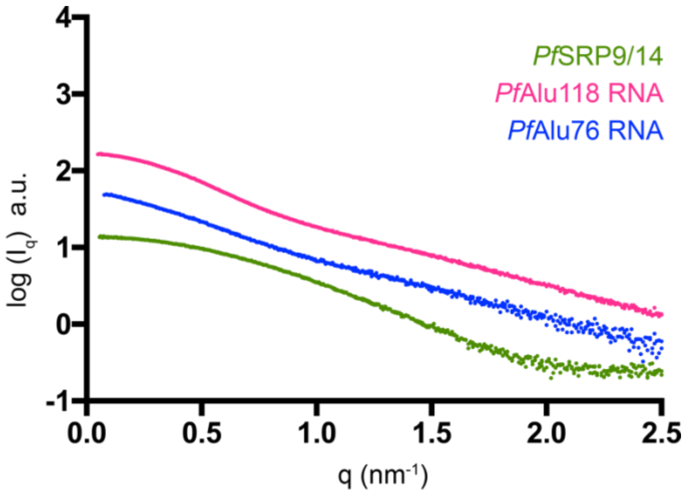

Supplementary Fig. 5: SAXS profiles of the *P. falciparum* Alu domain protein and RNA components
SAXS profiles of *Pf*SRP9/14 (green), *Pf*Alu118 RNA (magenta) and *Pf*Alu76 RNA (blue) plotted as $\log(I_q)$ versus q. a.u. represents arbitrary units.

Supplementary Figure 6

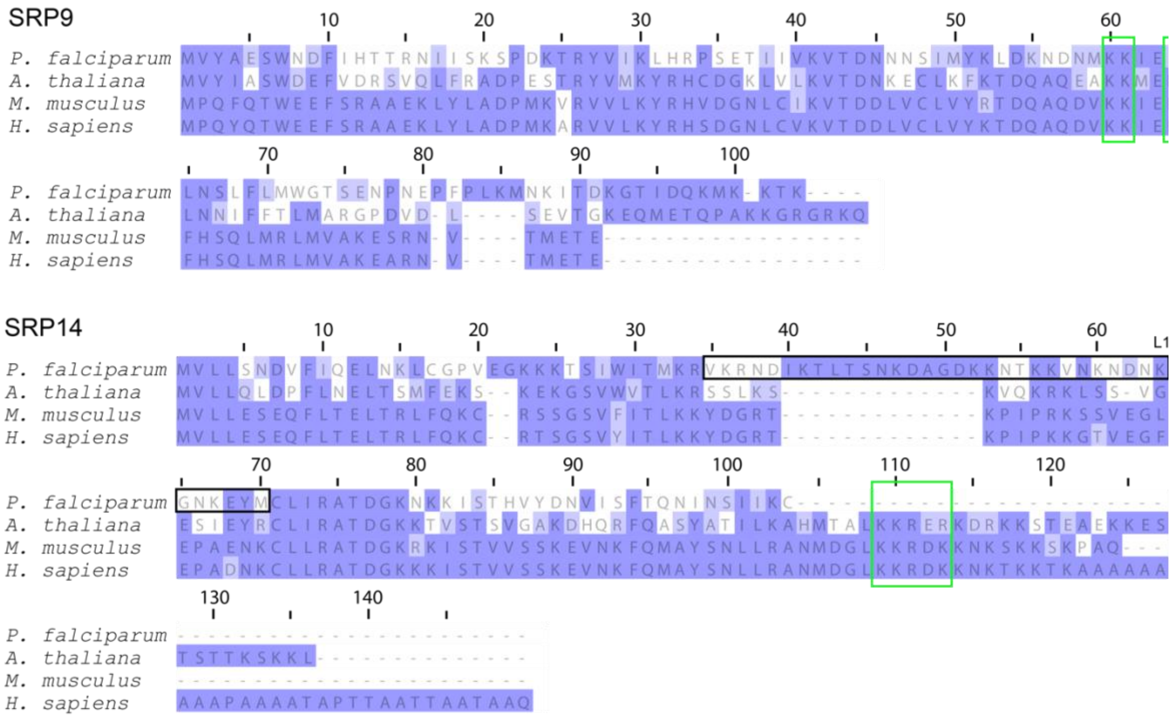

Supplementary Fig. 6: Multiple sequence alignment of SRP9/14
Cross-species alignment of SRP9 and SRP14 are shown. Residues are colored according to Blosum62 based coloring in Jalview[4]. Residues in *Hs*SRP9/14 shown to be required for elongation arrest activity are boxed in green[5,6]. The extended loop L1 in *P. falciparum* SRP14 is boxed in black.

Supplementary Figure 7

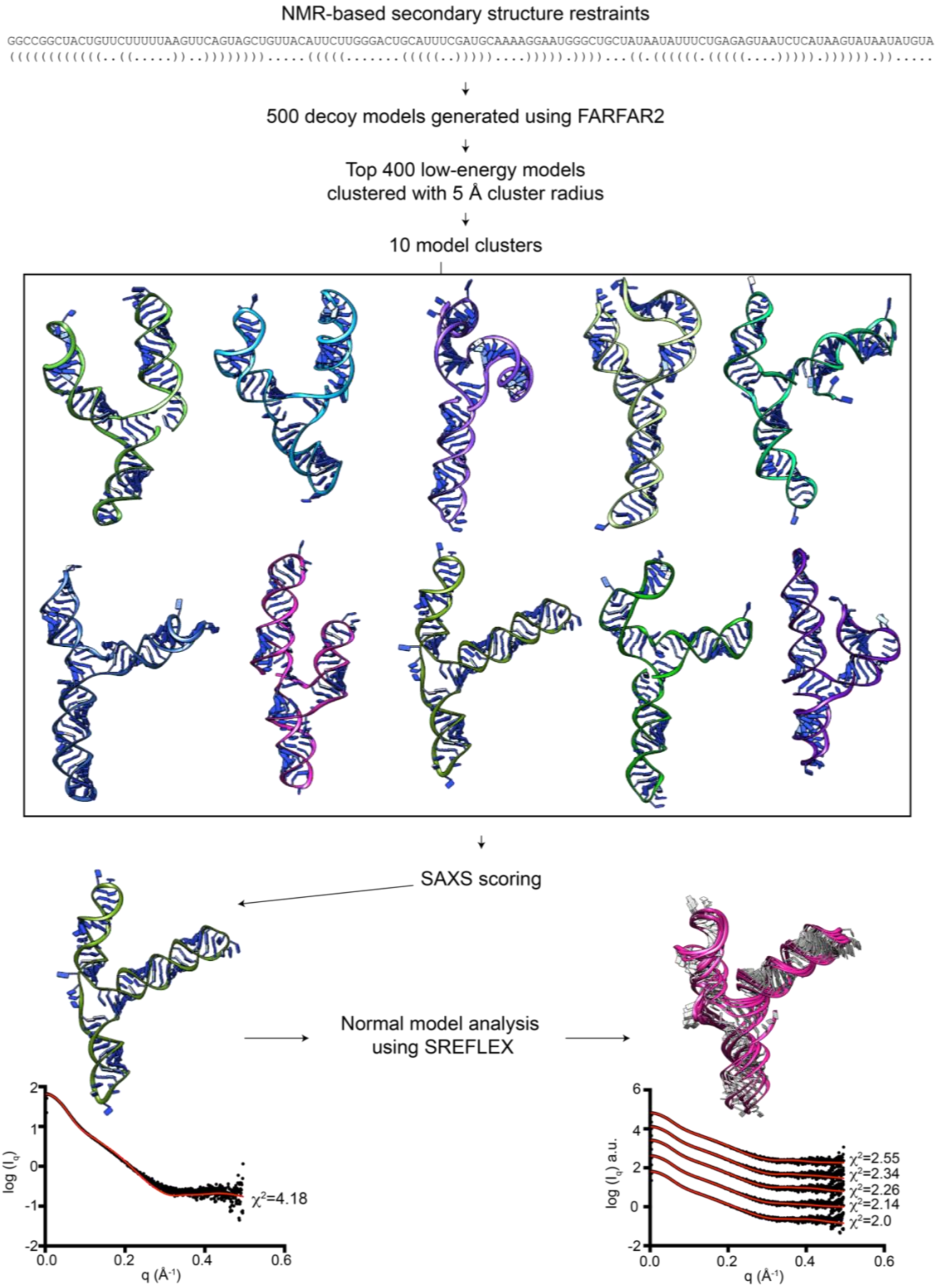

Supplementary Fig. 7: Schematic of NMR-SAXS based RNA modeling using the FARFAR2 webserver

NMR-based secondary structure restraints are used as input to the FARFAR2[7] modeling webserver to generate 500 decoy models, of which 400 lowest energy models are used for clustering to produce 10 model clusters. The clusters are further scored using SAXS and the best scoring cluster is further subjected to normal mode analysis using SREFLEX[8] to generate 5 models, which better fit the experimental SAXS data. SAXS-based scoring is performed using CRYSOL[9]. χ^2 fitting values of the experimental SAXS and back-calculated SAXS profiles of the different models are presented.

Supplementary Figure 8

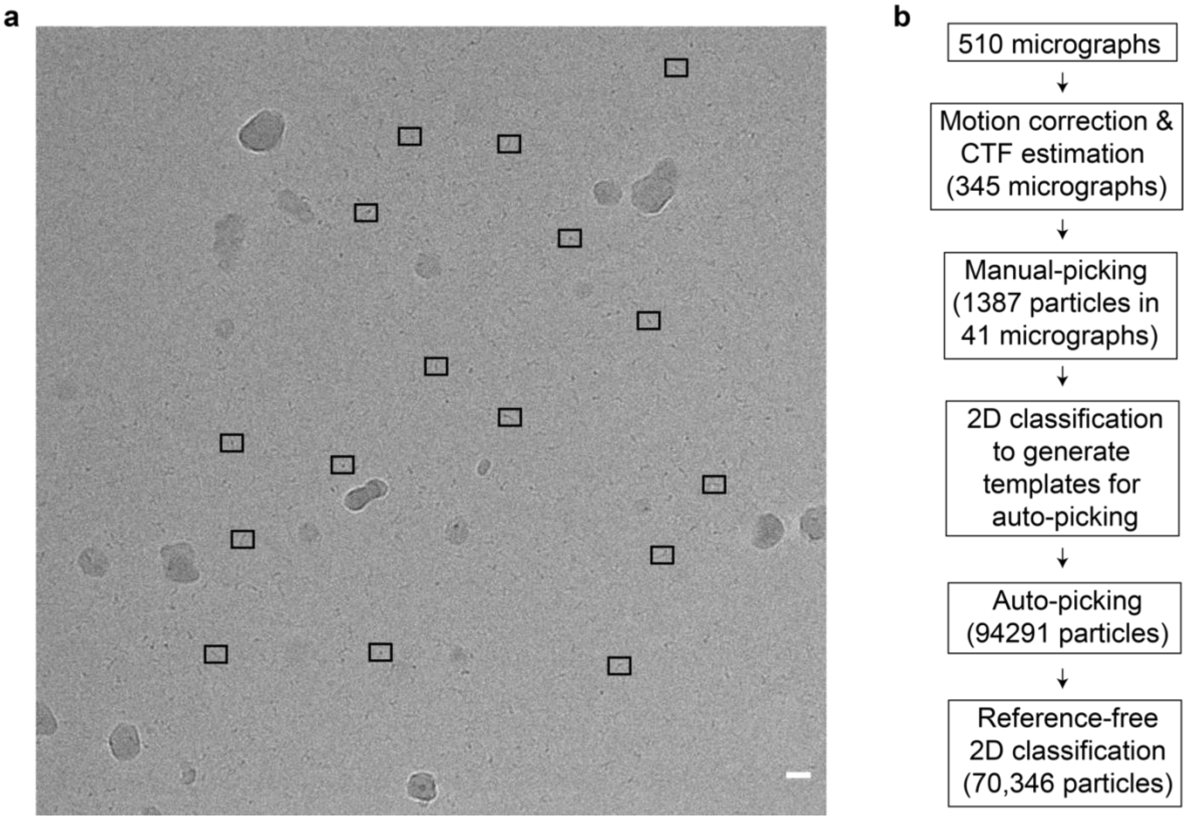

Supplementary Fig. 8: Cryo-EM processing workflow
a A motion-corrected EM-micrograph with representative *Pf*Alu118 RNA particles boxed. The scale bar represents 200 Å. **b** Workflow of cryo-EM data processing in RELION 3.1.

Supplementary Figure 9

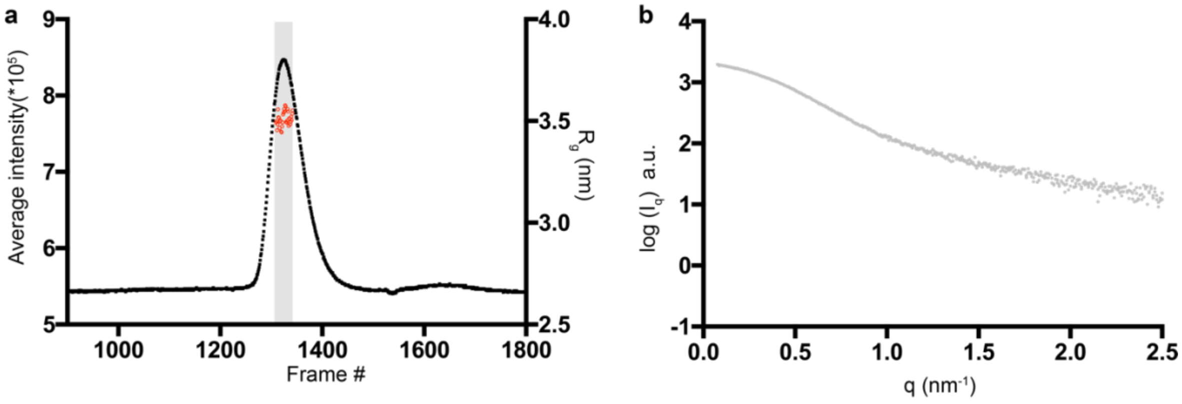

Supplementary Fig. 9: SAXS analysis of the *Pf*Alu domain
a SEC-SAXS profile of *Pf*Alu domain plotted according to frame number. Frames used for obtaining averaged data are highlighted and the radius of gyration is shown in red. **b** SAXS profile of *Pf*Alu domain plotted as log(I_q) versus q. a.u. represents arbitrary units.

Supplementary Table 1

Sample	Average experimental molecular weight (kDa)	Theoretical molecular weight (kDa)
*Pf*SRP9/14	24±0.5	24.1
*Pf*Alu118 RNA	33.2±0.1	38.6
*Pf*Alu domain	55.6±0.1	62.7

Supplementary Table 1. SEC-MALS derived average molecular weight

Supplementary Table 2

Data collection parameters	*Pf*SRP9/14	*Pf*Alu76 RNA	*Pf*Alu118 RNA	*Pf*Alu domain
Instrument	BM29 (ESRF, Grenoble)	BM29 (ESRF, Grenoble)	BM29 (ESRF, Grenoble)	BM29 (ESRF, Grenoble)
Beam geometry (mm^2)	0.7×0.7	0.7×0.7	0.7×0.7	0.7×0.7
Wavelength (Å)	0.99	0.99	0.99	0.99
q range (nm^{-1})	0.03-5	0.03-5	0.03-5	0.03-5
Exposure time (s)	5s (10×0.5s)	10s (20×0.5s)	10s (20×0.5s)	1s ×2500 frames
Temperature (°C)	20	20	20	20
Concentration range measured (mg ml^{-1})	0.3 - 5	0.2 - 1.8	0.4 - 2.8	Unknown (SEC)
Concentration used (mg ml^{-1})	2.5	0.45	2.8	-
Structural parameters				
R_g (nm) (from $P(r)$)	2.1	3.35	3.33	3.5
R_g (nm) (from Guinier)	2.08±0.01	3.34±0.02	3.32±0.03	3.50±0.05
D_{max} (nm)	7.2	11.5	11.8	12
Porod volume estimate (nm^3) (from P(r))	40.45	38.31	63.3	119.47
Excluded volume estimate[§] (nm^3)	48	52	79	127
Molecular weight determination (kDa)				
From volume of correlation (V_c)	26.2	26.7	37.6	62.6
From Bayesian assessment [credibility interval], probability	23.7 [22.1, 24.7], 96.3%	28.9 [27.9, 32], 92.5%	37.7 [36.5, 40.7], 94.7%	62.4 [58.8,68], 93.6%
Calculated *MW* from sequence	24.1	24.9	38.6	62.7
Software employed				
Primary data reduction	BsxCuBE			
Data processing	PRIMUS	PRIMUS	PRIMUS	CHROMIXS, PRIMUS
Ab initio analysis	DAMMIF	DAMMIF	DAMMIF	MONSA
Validation, averaging and final refinement	DAMAVERDAM MIN	DAMAVER DAMMIN	DAMAVER DAMMIN	-
3D structure prediction	-	FARFAR2	FARFAR2	-
Flexibility modeling	-	SREFLEX	SREFLEX	-
Computation of model intensities	CRYSOL			
3D graphics representations	UCSF Chimera			

Supplementary Table 2: SAXS data collection, processing and modeling statistics

Supplementary Table 3

Microscope	Glacios
Detector	Falcon3
Magnification	92,000
Voltage (kV)	200
Electron exposure (e-/ Å^2)	63.68
Defocus range (µm)	-1.5 to -3.0
Pixel size (Å)	1.56
Symmetry imposed	C1
Initial particle images (no.)	94,291
Final particle images (no.)	70,346
Data collection software	EPU
Data processing software	RELION-3.1

Supplementary Table 3: Cryo-EM data collection and processing statistics for *Pf*Alu118 RNA

Supplementary Table 4

	Constraints			χ^2		
No. of models	*Pf*SRP9/14	*Pf*Alu118 RNA	*Pf*Alu domain	$\chi^2_{Pf\mathrm{SRP9/14}}$	$\chi^2_{PfAlu118\,RNA}$	$\chi^2_{PfAlu\,domain}$
1	Yes	Yes	Yes	0.99	1.45	1.29
1	Yes	-	Yes	0.902	-	1.22
1	-	Yes	Yes	-	1.4	1.47
10*	Yes	Yes	Yes	1.03	1.44	1.32

(*χ^2 averages of 10 independent runs are presented)

Supplementary Table 4: MONSA modeling of the *Pf*Alu domain

Supplementary References

1 Voorhees, R. M. & Hegde, R. S. Structures of the scanning and engaged states of the mammalian SRP-ribosome complex. *Elife* **4**, e07975 (2015).

2 Bousset, L. *et al.* Crystal structure of a signal recognition particle Alu domain in the elongation arrest conformation. *RNA* **20**, 1955-1962 (2014).

3 Rosenblad, M. A., Zwieb, C. & Samuelsson, T. Identification and comparative analysis of components from the signal recognition particle in protozoa and fungi. *BMC Genomics* **5**, 5 (2004).

4 Waterhouse, A. M., Procter, J. B., Martin, D. M., Clamp, M. & Barton, G. J. Jalview Version 2--a multiple sequence alignment editor and analysis workbench. *Bioinformatics* **25**, 1189-1191 (2009).

5 Lakkaraju, A. K., Mary, C., Scherrer, A., Johnson, A. E. & Strub, K. SRP keeps polypeptides translocation-competent by slowing translation to match limiting ER-targeting sites. *Cell* **133**, 440-451 (2008).

6 Mary, C. *et al.* Residues in SRP9/14 essential for elongation arrest activity of the signal recognition particle define a positively charged functional domain on one side of the protein. *RNA* **16**, 969-979 (2010).

7 Watkins, A. M., Rangan, R. & Das, R. FARFAR2: Improved De Novo Rosetta Prediction of Complex Global RNA Folds. *Structure* **28**, 963-976 (2020).

8 Panjkovich, A. & Svergun, D. I. Deciphering conformational transitions of proteins by small angle X-ray scattering and normal mode analysis. *Phys Chem Chem Phys* **18**, 5707-5719 (2016).

9 Franke, D. *et al.* ATSAS 2.8: a comprehensive data analysis suite for small-angle scattering from macromolecular solutions. *J Appl Crystallogr* **50**, 1212-1225 (2017).
