# Supplementary material for: Structural analysis of the SRP Alu domain from Plasmodium falciparum reveals a non-canonical open conformation
Source: Commun Biol. 2021 May 20;4:600. doi: 10.1038/s42003-021-02132-y (PMC8137916; doi:10.1038/s42003-021-02132-y)
Supplement: Supplementary file 4 — Reporting Summary [file 42003_2021_2132_MOESM4_ESM.pdf]

nature research

Corresponding author(s): Irmgard Sinning

Last updated by author(s): Feb 18, 2021

Reporting Summary

Nature Research wishes to improve the reproducibility of the work that we publish. This form provides structure for consistency and transparency in reporting. For further information on Nature Research policies, see our Editorial Policies and the Editorial Policy Checklist.

Statistics

For all statistical analyses, confirm that the following items are present in the figure legend, table legend, main text, or Methods section.

n/a	Confirmed	
☐	☒	The exact sample size (*n*) for each experimental group/condition, given as a discrete number and unit of measurement
☒	☐	A statement on whether measurements were taken from distinct samples or whether the same sample was measured repeatedly
☒	☐	The statistical test(s) used AND whether they are one- or two-sided<br>*Only common tests should be described solely by name; describe more complex techniques in the Methods section.*
☒	☐	A description of all covariates tested
☒	☐	A description of any assumptions or corrections, such as tests of normality and adjustment for multiple comparisons
☒	☐	A full description of the statistical parameters including central tendency (e.g. means) or other basic estimates (e.g. regression coefficient) AND variation (e.g. standard deviation) or associated estimates of uncertainty (e.g. confidence intervals)
☒	☐	For null hypothesis testing, the test statistic (e.g. *F*, *t*, *r*) with confidence intervals, effect sizes, degrees of freedom and *P* value noted<br>*Give P values as exact values whenever suitable.*
☒	☐	For Bayesian analysis, information on the choice of priors and Markov chain Monte Carlo settings
☒	☐	For hierarchical and complex designs, identification of the appropriate level for tests and full reporting of outcomes
☒	☐	Estimates of effect sizes (e.g. Cohen's *d*, Pearson's *r*), indicating how they were calculated

Our web collection on statistics for biologists contains articles on many of the points above.

Software and code

Policy information about availability of computer code

Data collection	Topspin v3.2 (Bruker), EPU v2.8 (ThermoFischer), BsxCuBE beamline software (ESRF), MO.Control Software
Data analysis	Softwares used: Pymol (version 2.3.1), Chimera (version 1.12), CcpNmr Analysis (version 2.4), GraphPad Prism (version 6), PRIMUS and CHROMIXS (ATSAS version 2.8.3), SREFLEX (ATSAS version 3.0.2), CRYSOL (ATSAS version 3.0.2), DAMMIN/F (ATSAS version 3.0.2), MO.Affinity analysis, Astra 6 software (Wyatt Technology)

For manuscripts utilizing custom algorithms or software that are central to the research but not yet described in published literature, software must be made available to editors and reviewers. We strongly encourage code deposition in a community repository (e.g. GitHub). See the Nature Research guidelines for submitting code & software for further information.

Data

Policy information about availability of data

All manuscripts must include a data availability statement. This statement should provide the following information, where applicable:

- Accession codes, unique identifiers, or web links for publicly available datasets
- A list of figures that have associated raw data
- A description of any restrictions on data availability

NMR imino resonance assignments have been deposited to the BMRB for PfAlu RNA helices H3, H4 and H5 with accession codes: 50570, 50571 and 50572, respectively. SAXS data for PfSRP9/14 heterodimer, PfAlu118 RNA, PfAlu76 RNA and PfAlu118 RNA in complex with PfSRP9/14 heterodimer have all been deposited to the SASBDB with accession codes: SASDK24, SASDK34, SASDK44 and SASDK54 respectively.

Field-specific reporting

Please select the one below that is the best fit for your research. If you are not sure, read the appropriate sections before making your selection.

- [x] Life sciences
- [ ] Behavioural & social sciences
- [ ] Ecological, evolutionary & environmental sciences

For a reference copy of the document with all sections, see nature.com/documents/nr-reporting-summary-flat.pdf

Life sciences study design

All studies must disclose on these points even when the disclosure is negative.

Sample size	For MST data, two or three biological replicates were performed.
Data exclusions	No data were excluded from analysis.
Replication	MST binding data were performed in duplicates or triplicates.
Randomization	There was no allocation of groups done in this study and so no Randomization was required.
Blinding	There was no allocation of groups done in this study and so no Randomization was required.

Reporting for specific materials, systems and methods

We require information from authors about some types of materials, experimental systems and methods used in many studies. Here, indicate whether each material, system or method listed is relevant to your study. If you are not sure if a list item applies to your research, read the appropriate section before selecting a response.

Materials & experimental systems

n/a	Involved in the study	
x		Antibodies
x		Eukaryotic cell lines
x		Palaeontology and archaeology
x		Animals and other organisms
x		Human research participants
x		Clinical data
x		Dual use research of concern

Methods

n/a	Involved in the study	
x		ChIP-seq
x		Flow cytometry
x		MRI-based neuroimaging
